# A Robust Flow-Through Platform for Organic Contaminant Removal

**DOI:** 10.1016/j.xcrp.2020.100296

**Published:** 2021-01-20

**Authors:** Long Chen, Akram N. Alshawabkeh, Shayan Hojabri, Meng Sun, Guiyin Xu, Ju Li

**Affiliations:** 1 Department of Civil and Environmental Engineering, Northeastern University, Boston, MA 02115, USA; 2 Department of Chemical and Environmental Engineering, Yale University, New Haven, CT 06520-8286, USA; 3 Department of Nuclear Science and Engineering, Massachusetts Institute of Technology, Cambridge, MA 02139, USA; 4 Department of Materials Science and Engineering, Massachusetts Institute of Technology, Cambridge, MA 02139, USA; 5 Lead Contact

## Abstract

Achieving the greatest cleanup efficiency with minimal footprint remains a paramount goal of the water treatment industry. Toxic organic compounds threaten drinking water safety and require effective pretreatment. Hydroxyl radicals produced by the Fenton process (Fe^2+^/H_2_O_2_) destroy organic contaminants based on their strong oxidation potential. An upgraded reaction using solid catalysts, referred to as the Fenton-like process, was recently adopted to avoid the ferric sludge generation during the conventional Fenton process. However, most heterogeneous Fenton-like catalysts operate optimally at pH 3–5 and quite weakly in near-neutral water bodies. Here, we evaluate the feasibility of an electrolytically localized acid compartment (referred to as the *Ella* process) produced by electrochemical water splitting under flow-through conditions to facilitate the Fenton-like process. The *Ella* process boosts the activity of an immobilized iron oxychloride catalyst >10-fold, decomposing organic pollutants at a high flow rate. The robust performance in complex water bodies further highlights the promise of this platform.

## Abstract (summary)

Clean water is a sustainable development goal of the United Nations, but it is threatened by toxic organic pollutants. Chen et al. aim to upgrade an advanced oxidation water treatment unit, using electricity to generate the strong acidity required by the Fenton process, providing a path to green water treatment.

## Graphical Abstract

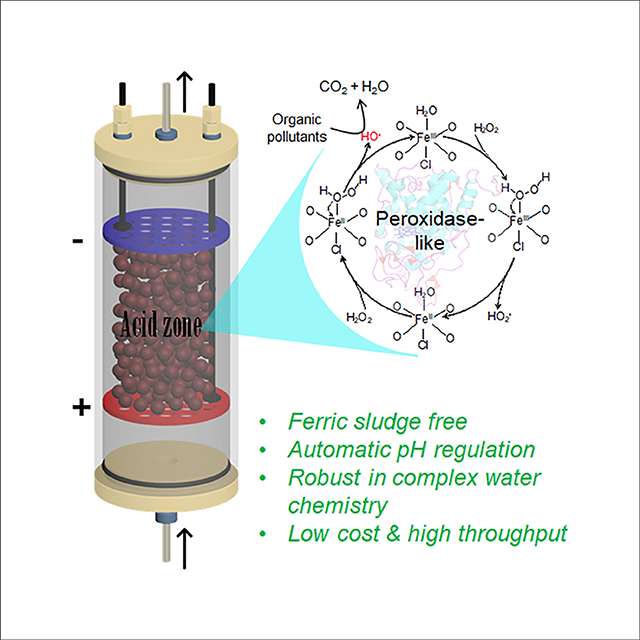

## INTRODUCTION

Various toxic organic compounds in the environment pose considerable threats to human health and the ecosystem. While efficient in providing clean drinking water, many advanced water treatment facilities have high capital and running costs that remain the principal barriers to implementation in developing countries.^1^ The classic Fenton process, which transforms hydrogen peroxide (H_2_O_2_) into hydroxyl radicals (HO^•^) under the ferrous ions (Fe^2+^) catalysis, is an affordable and proven water treatment technique.^2^ HO^•^ is the second most oxidative species in nature after fluorine, with a redox potential of 2.73 V versus normal hydrogen electrode^2^, and could non-selectively destroy recalcitrant organic contaminants in water.^3,4^ By coupling with other water treatment units, such as coagulation, membrane filtration, or microbial degradation, the Fenton process can be adapted for various treatments of bodies of water.^5–7^ Fruitful progress has been achieved to date in Fenton chemistry for water treatment.^8–12^ In particular, *in situ* synthesis of H_2_O_2_ from H_2_ and O_2_ under noble metal catalysis^13^ and H^+^ and O_2_ under photochemical^14^ or electrochemical catalysis^11^ are sustainable routes to reduce the chemicals cost. However, the formation of ferric sludge continues to be a challenge limiting implementation of the Fenton process. The sludge is formed when water pH is improved to approximately neutral after oxidation, and its toxicity stems from the adsorbed residual compounds in treated water.^15^ Treatment of the ferric sludge requires substantial chemical and manpower costs—10% to 50% of the overall operating costs in a water treatment plant.^16,17^

Heterogeneous solid catalysts could overcome the drawback of ferric sludge formation. Both naturally occurring clays/minerals and transition metal-centered designer composites have been extensively explored as Fenton-like catalysts.^8,18–21^ These catalysts have noteworthy structural elegance in the unique coordination environment of their metal cores, which stabilizes the metal-H_2_O_2_ complex transition state and facilitates the electron transfer *inter se*.^22^ Furthermore, the turnover of metal atoms on the catalyst surface occurs via a peroxidase-mimicking mechanism (see Figure S1 for peroxidase structure),^23^ akin to the Haber-Weiss reaction of homogeneous Fenton chemistry.^24^ The pH dependence of heterogeneous Fenton-like catalysts is generally less strict than that of free iron ions, the latter of which is the most effective at pH 2.8–3.5.^19^ Nevertheless, most canonical Fenton-like catalysts favor acidic pH 3–5 (Table S1), and the catalyst turnover frequency (TOF) can be reduced by up to 100-fold under neutral conditions.^25,26^ This is due to surface metal-OH complexes forming at higher pH values and repelling H_2_O_2_ away from the exposed active sites. However, pH regulation of near-neutral drinking water bodies is challenging, especially if the water contains high carbonate alkalinity; furthermore, the acidification of contaminated groundwater for *in situ* water treatment could release undesired metal ions from aquifers.^27,28^ These concerns severely compromise the potential use of heterogeneous Fenton-like catalysts in the water treatment industry.

Flow electrochemistry holds great promise for automatic pH regulation to support water treatment, wherein OH^−^ and H^+^ generated from the cathode and anode, respectively, are redistributed based on ion migration, dispersion, and hydraulic flux.^29^ Here, we propose an electrolytically localized acidic-compartment (*Ella*) process to regulate solution pH for heterogeneous Fenton-like reactions, building on our extensive experiences in flow electrochemistry. The *Ella* process has a small footprint because it turns pH-neutral influent into acidic solution in between the electrodes, and zones beyond that are back to neutral as the influent. Combined with heterogeneous Fenton-like catalysts, the *Ella* process demonstrated excellent performance under long-term use, high flow rate, and complex water chemistry, manifesting the robustness of this coupled platform for recalcitrant organic compounds removal.

## RESULTS AND DISCUSSION

### Design of a Flow-Through Water Treatment Platform

**Figure 1.**
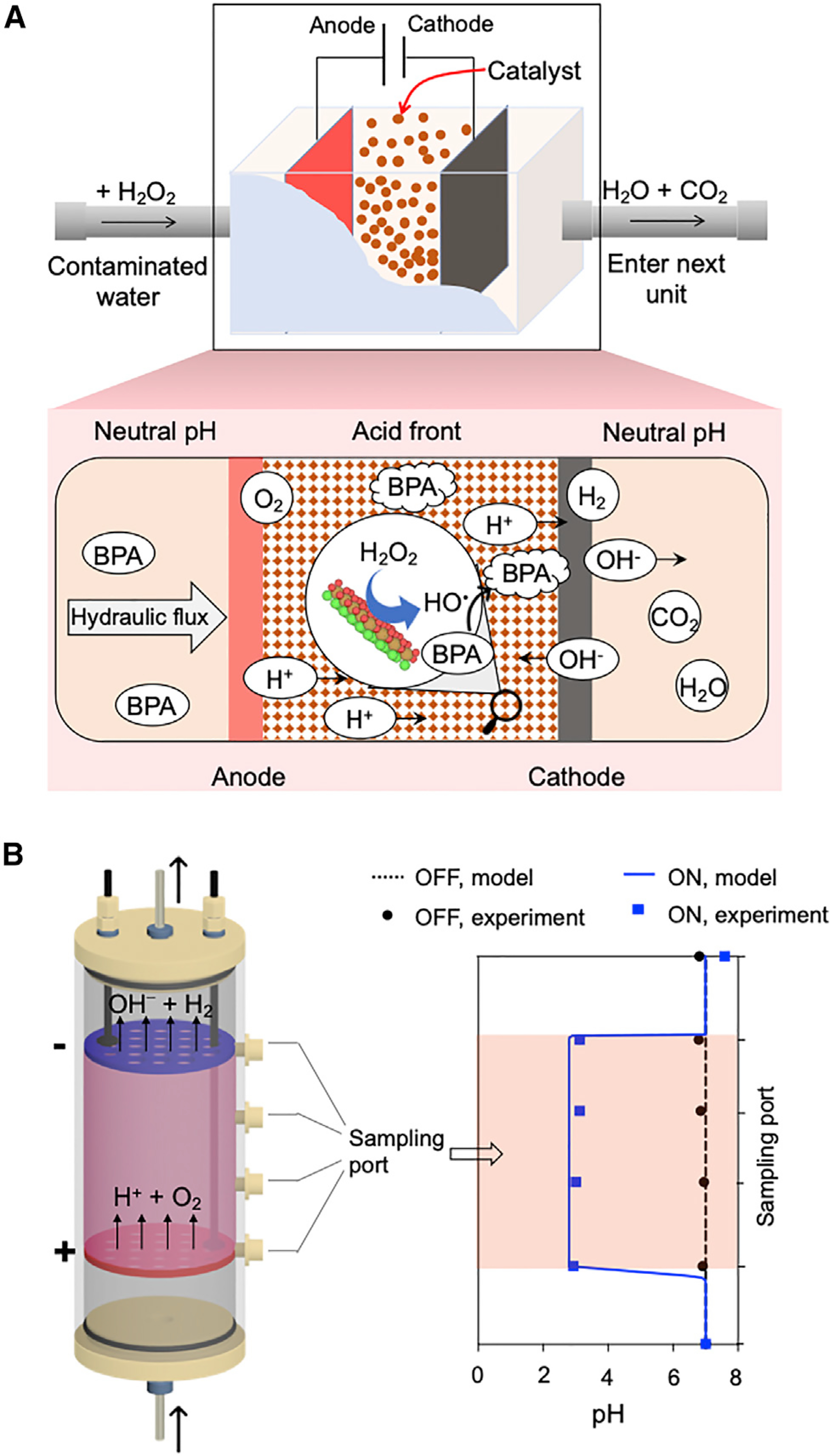
*Ella* Process-Based Flow-Through Water Treatment Platform (A) Schematic representation of the flow-through platform. Placement of an anode upstream of a cathode produces an acid front, which promotes the activity of immobilized Fenton-like catalyst. The H_2_O_2_ is *in situ* transformed into HO^•^ radical under catalysis, leading to oxidative mineralization of organic contaminant into CO_2_ and H_2_O. Bisphenol A (BPA) is used as a target compound for illustration purpose. (B) Automatic pH regulation by the *Ella* process. The left panel depicts the setup configuration, and the right panel indicates the pH variation profile along the column axis as the applied electric current was turned off and on. Arrows indicate flow direction. + and − denote anode and cathode, respectively.

The *Ella* process was used to regulate the pH of heterogeneous Fenton-like catalysts for water treatment with desired acidity. To this end, immobilized catalysts must be transferred to the acidic zone, whereby H_2_O_2_ is rapidly transformed into oxidizing HO^•^ radicals, leading to the instant degradation of organic compounds in the local vicinity (Figure 1A). This coupled water treatment process is categorized as an electro-Fenton-like (EFL) platform in this study. The flow-through treatment method is especially favored over the batch reaction mode for industry applications, since a water treatment plant treats high volumes of contaminated water at a fast pace. It is worth of noting that the EFL platform can take advantage of the intermittent solar and wind electricity, often with near-zero or even negative prices.

The contaminants treatment efficiency by the EFL platform is in the main determined by the reaction during the transport through the catalyst column—that is, longer retention times and higher catalyst and H_2_O_2_ concentrations tend to result in more complete contaminant removal. Therefore, the key aspects to warrant the success of the EFL platform are to promote HO^•^ generation and allow sufficient reaction time within the column.

### pH Regulation by the *Ella* Process

As a proof of concept, a benchtop flow-through device was manufactured for our EFL platform. A set of stable mixed metal oxide electrodes were installed 9 cm apart in a vertically anchored acrylic flow-through column device (15 cm × 5 cm outer diameter [OD]). The column was filled with clean silica sand (0.15–0.6 mm) to maintain a laminar flow. The combination of a 100-mA electric current and a 15-mL/min hydraulic flow rate was applied to the pH-neutral solution passing through the column, and solution pHs at various locations along the column were measured (Figure 1B). The solution was homogeneously neutral without electric current; however, by turning on the 100-mA electric current, an acidic pH of 2.93–3.12 between the anode and cathode was automatically attained at steady state. The electrochemically created acidity is suitable for most Fenton-like catalysts. The effluent pH measured as 7.59 was close to the influent value (pH 7), showing minimal effect on the treated water pH. A reactive transport model (see Supplemental Experimental Procedures) that accounts for advection, hydrodynamic dispersion, and ion migration was used to simulate the pH profile.^30^ Modeling results indicate that the theoretical acidity of the confined space between electrodes using the above stated experimental settings is pH 2.8. The slight discrepancy in pH between experimental results and modeled value is possibly due to the fact that the current efficiency of water electrolysis was not 100%.

### Immobilization of Fenton-like Catalyst

Iron oxychloride (FeOCl) was reported to possess extraordinary Fenton-like catalytic activity with high fidelity and can be mass produced.^22,31^ Characterizations of the synthetic FeOCl nanocatalyst produced via the calcination of FeCl_3_•6H_2_O were shown in Figures S2 and S3. Bisphenol A (BPA) was used as the primary pollutant to illustrate FeOCl catalytic degradation performance, as BPA is an environmental estrogen that disrupts the human endocrine system upon exposure.^32^ A total of 10 μM BPA was rapidly degraded by the FeOCl/H_2_O_2_ reaction. However, the addition of ethanol, a strong HO^•^ radical quencher,^33^ competed with BPA for the produced HO^•^ radical reservoir and fully inhibited BPA removal by the FeOCl/H_2_O_2_ reaction (Figure S4), demonstrating that the degradation of BPA was via HO^•^ radical (Note S1). It was further found that FeOCl showed optimal activity at pH 3, ~9.3-fold higher than that at pH 7 in terms of HO^•^ radical yield.

Density functional theory (DFT) calculation was harnessed to study the catalysis mechanism of the FeOCl/H_2_O_2_ reaction. It was revealed that the reaction is accomplished via two electron-transfer processes (Figure S5). In the first step, Fe^III^OCl is reduced by H_2_O_2_ into Fe^II^OCl. In the second step, the derived Fe^II^OCl decomposes H_2_O_2_ into HO^•^ radical via homolytic cleavage. The readily reducible nature of unsaturated Fe atoms on the exposed (100) surface of FeOCl crystals allows rapid turnovers of Fe^III/II^ to catalyze the H_2_O_2_ transformation. Specifically, the energy barrier of reducing Fe^III^ to Fe^II^ on FeOCl crystals by H_2_O_2_ was determined as 0.235 eV (Figure S6), whereas that of hematite (Fe_2_O_3_, a rhombohedral Fenton-like catalyst) is 0.76 eV as a comparison.^34^

It is, however, of great concern that the microcatalyst particles are subject to fluid transport, resulting in potential draining from the silica sand pores within the column device. For instance, by packing well-mixed FeOCl and silica sand particles into the column (Figure S7; Note S2), the overall catalyst activity decayed by 36% after 8 h due to hydraulic erosion (Figure S8). Another immobilization strategy of cross-linking FeOCl with alginate hydrogel was attempted for the effective retention of FeOCl nanoparticles (Figure S9; Note S3).^35^ However, the resulting FeOCl/alginate composite showed only 1% activity compared to the same amount of unimmobilized FeOCl catalyst (Figure S10; Note S4), delivering poor in-reactor performance under high flow rates (Figures S11 and S12; Note S5). This is due to a large fraction of FeOCl particles becoming buried inside the hydrogel of the FeOCl/alginate composite and not being effectively accessible by H_2_O_2_ molecules.

**Figure 2.**
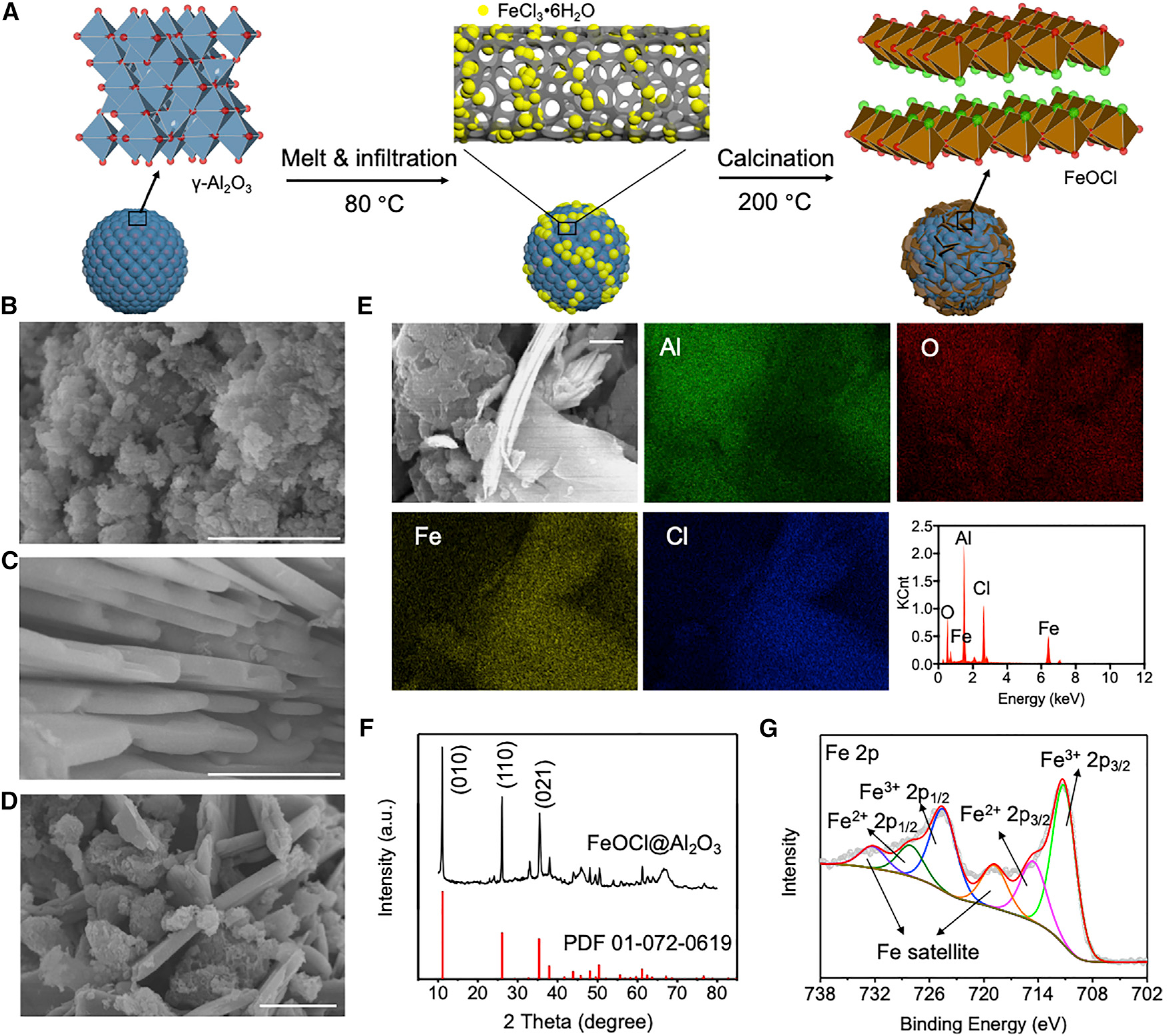
Immobilization of FeOCl onto γ-Al_2_O_3_ Support (A) Illustration of melt infiltration strategy. (B–D) SEM images of γ-Al_2_O_3_ (B), FeOCl (C), and FeOCl/Al_2_O_3_ (D). (E) EDX element mapping and analysis of FeOCl/Al_2_O_3_. (F) XRD pattern of FeOCl/Al_2_O_3_. (G) Fe 2p deconvolution spectra of XPS analysis. Scale bars indicate 1 μm.

To solve this problem, FeOCl nanoparticles were alternatively immobilized on a porous γ-Al_2_O_3_ support via a melt infiltration method^36^ (Figure 2A), with the belief that this approach offers maximum FeOCl catalytic sites for H_2_O_2_ molecules. Field emission scanning electron microscopy (FESEM) images clearly revealed the dispersion of FeOCl nanosheets on the amorphous γ-Al_2_O_3_ support in the synthesized FeOCl/Al_2_O_3_ composite (Figures 2B–2D), with a surface area of 206 m^2^/g (Table S2). The compositional distribution of the FeOCl/Al_2_O_3_ was investigated with energy-dispersive X-ray spectroscopy (EDX) (Figure 2E). EDX analysis identified Al, O, Fe, and Cl elements on the FeOCl/Al_2_O_3_ composite. Elemental mapping results suggested that Al and Fe elements were separately located, in agreement with the overlay structure of the FeOCl/Al_2_O_3_ composite. Cl element exhibited a consistent pattern with Fe element on the FeOCl surface, while O element was uniformly distributed on the FeOCl/Al_2_O_3_ composite. X-ray diffraction (XRD) of the FeOCl/Al_2_O_3_ composite disclosed several characteristic diffraction peaks (2θ) at 11.2°, 26.1°, and 35.4°, which respectively belongs to the (010), (110), and (021) planes of the orthorhombic FeOCl crystal (Powder Diffraction File [PDF]: 01–072-0619) (Figure 2F). The chemical states of compositional elements were studied with X-ray photoelectron spectroscopy (XPS).^37^ In particular, deconvoluted Fe 2p spectrum showed that Fe^3+^ 2p_1/2_ (724.6 eV) and 2p_3/2_ (711.2 eV) were dominant over Fe^2+^ 2p_1/2_ (728.2 eV) and 2p_3/2_ (714.6 eV) (Figure 2G), which is consistent with the unimmobilized FeOCl crystal (Figure S3). XPS analyses of other elements in the FeOCl/Al_2_O_3_ composite and the γ-Al_2_O_3_ support are shown in Figures S13 and S14. Overall, the results above suggested that the morphology and electronic properties of the FeOCl crystal remained intact after immobilization on the γ-Al_2_O_3_ support.

**Figure 3.**
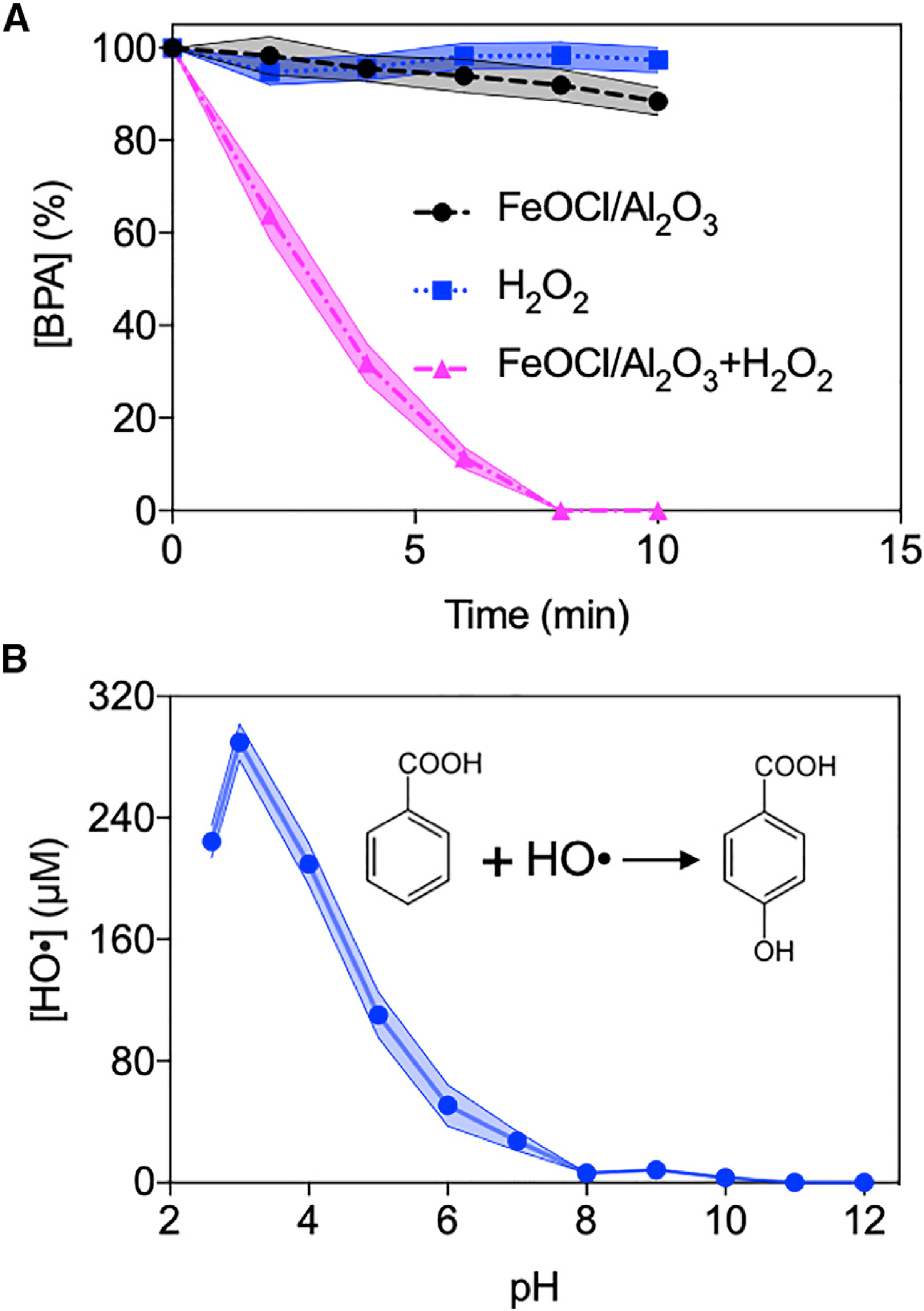
Catalytic Activity of FeOCl/Al_2_O_3_ Composite (A) BPA degradation by FeOCl/Al_2_O_3_ and H_2_O_2_ reaction and controls. Reactions were performed at pH 3. (B) Quantification of generated HO^•^ radical by FeOCl/Al_2_O_3_ and H_2_O_2_ reaction at different pH after reaction for 30 min. Inset shows the stoichiometric oxidation of benzoic acid for HO^•^ radical yield determination. Shaded area indicates the error range of obtained data.

Activity test results of the synthetic FeOCl/Al_2_O_3_ composite are shown in Figure 3. It was found that 10 μM BPA was completely degraded in 10 min by 0.2 g/L FeOCl/Al_2_O_3_ and 10 mM H_2_O_2_ at pH 3, while the FeOCl/Al_2_O_3_ composite or H_2_O_2_ alone led to negligible BPA removals. Furthermore, under the stated conditions, FeOCl/Al_2_O_3_-mediated Fenton-like reaction produced the most remarkable amount of HO^•^ radicals at pH 3 (i.e., 289.8 μM after reaction for 30 min), the acidity of which could be readily achieved by the *Ella* process. The catalytic performance of the FeOCl/Al_2_O_3_ composite showed a pattern consistent with that of the unimmobilized FeOCl nanoparticle (Figure S4), indicating that the γ-Al_2_O_3_ support played no role in H_2_O_2_ transformation.

### High Performance of the Electro-Fenton-like Platform

**Figure 4.**
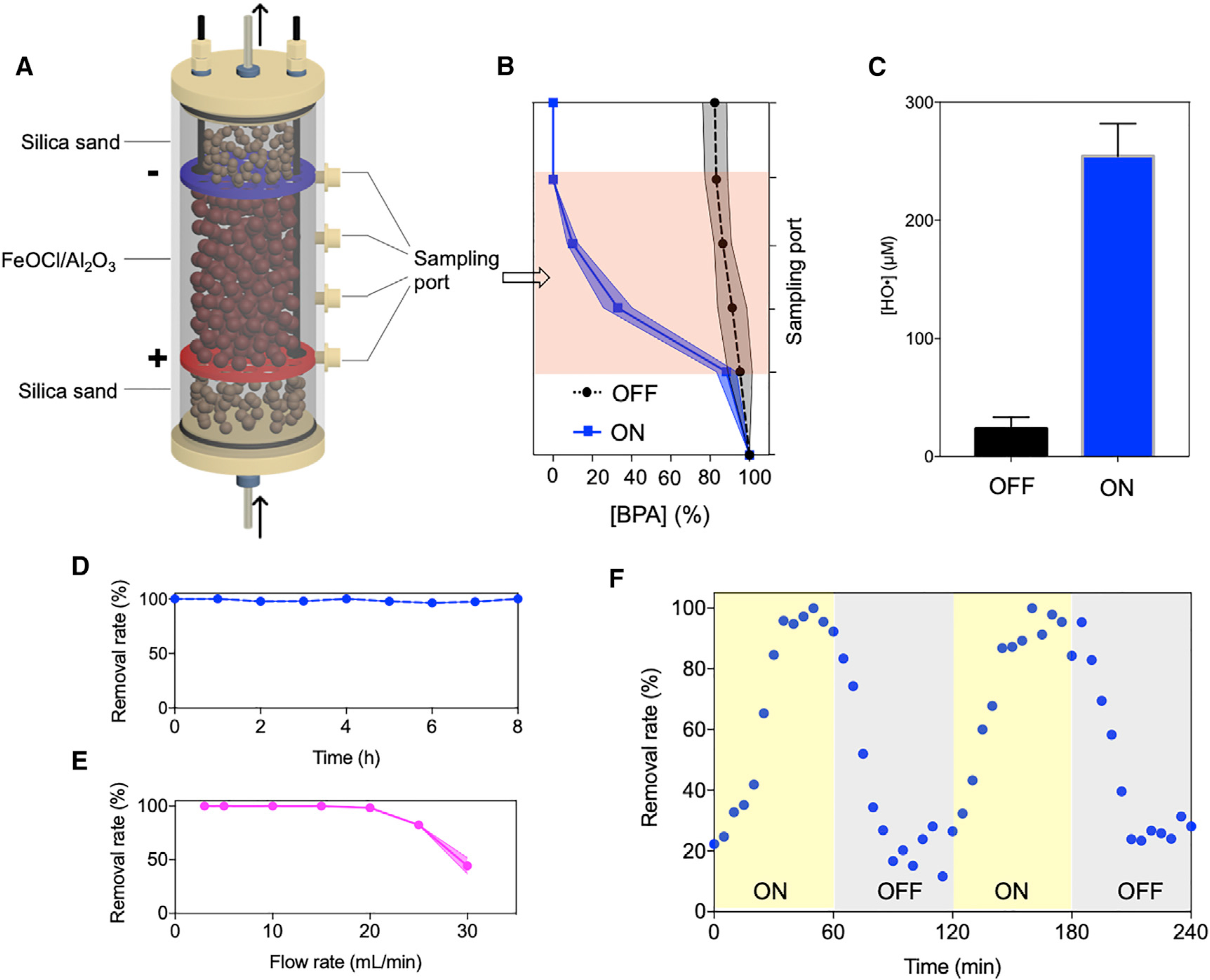
Performance of Electro-Fenton-like Platform (A) Illustration of column device components. (B and C) BPA removals from each sampling port (B), and (C) quantification of generated HO^•^ radical by electro-Fenton-like platform as the electric current was turned off and on. (D–F) Stability test (D), (E) tolerance of flow rate, and (F) electricity responsiveness assay of the electro-Fenton-like platform. Influent flow rate was subjected to change for (E). Arrows indicate flow direction. + and − denote anode and cathode, respectively. Data in (C) are represented as means G SEMs. Shaded areas in (B), (D), and (E) indicate the error range of obtained data.

The synthetic FeOCl/Al_2_O_3_ composites were transferred to the space between electrodes of the column to use the acidity produced by the *Ella* process (Figure 4A). Neutral solutions containing 10 mM H_2_O_2_ oxidant and 10 μM BPA contaminant were pumped through the column at a rate of 15 mL/min, and BPA removals along the hydraulic flux direction were measured (Figure 4B). Low BPA removal from the effluent (17.6%) was observed without electric current, due to the weak activity of FeOCl/Al_2_O_3_ under neutral condition. However, after a 100-mA electric current was applied to the electrodes, the gradual degradation of BPA took place as measured from the sampling ports, and BPA removal reached 100% in the effluent. Direct BPA degradation by electrodes was ruled out based on the control experiment without the FeOCl/Al_2_O_3_ catalyst (Figure S15). In addition, the presence of 10 mM ethanol as the HO^•^ radical scavenger almost fully quenched BPA removal (Figure S15), suggesting that *in situ*-produced HO^•^ radicals accounted for BPA degradation. As shown in Figure S16, the HO^•^ radicals generated by the EFL platform could also non-selectively degrade other organic contaminants, including drugs (ibuprofen and carbamazepine), herbicide (atrazine), pesticide and drug precursors (4-chlorophenol and 4-nitrophenol), and recalcitrant dyes (rhodamine B, reactive blue 19, and orange II). The operating process to treat a dye solution may be viewed in more detail in Video S1. It was further determined that the yield of HO^•^ radical produced by the platform when the 100-mA electric current was turned off and on was 23.5 and 254.4 μM, respectively (Figure 4C). This 10.8-fold increase in HO^•^ radical production was attributed to the acidic environment created by the *Ella* process, which boosted the FeOCl/Al_2_O_3_ catalyst activity. The results indicate that the EFL platform, by coupling a heterogeneous Fenton-like catalyst and the *Ella* process in a flow-through column device, is effective for the removal of organic contaminants.

The long-term stability of BPA removal by the EFL platform was tested (Figure 4D). Over the course of 8-h reactions, laminar fluid transport was maintained, and the BPA removal steadily approached 100%. Clearly, a duration of 8 h is not enough for practical industry application, but here is used as a proof of concept to demonstrate the durability of our system at the current stage. Future studies will include longer tests of up to weeks. BPA removal by this platform under different hydraulic flow rates was also measured (Figure 4E). A high flow rate leads to shortened contact time among H_2_O_2_ molecules, the catalyst surface, and the organic contaminant, which reduces HO^•^ radical yield and compromises contaminant removal. Over 95% of 10 μM BPA could be removed from the effluent at a flow rate of <20 mL/min, whereas the BPA removal rate decreased to 82.4% and 44.4% at 25 and 30 mL/min, respectively. The tolerance of flow rate by using FeOCl/Al_2_O_3_ in this design was greatly superior to that using FeOCl crosslinked on alginate hydrogel as a catalyst (Figure S12), primarily due to the abundant catalytic sites exposed on the surface of the FeOCl/Al_2_O_3_ composite.

The responsiveness of BPA removal by the EFL platform to the electric current was monitored by repeatedly turning the electric power supply on and off, with a 60-min interval. As shown in Figure 4F, BPA removal significantly increased after the 100-mA electric current was turned on for 15 min, and approached 100% after 40 min. As the power was turned off, BPA removal gradually decreased during the first 30 min, from 84.3%–92.3% to 16.7%–23.9%, and was steady afterward. The non-instant response of BPA removal to electricity was presumably because of the slow accumulation and the desorption of protons. The results demonstrated that electricity is a critical governor of contaminant removal by our developed platform. In addition, the observed response time in this study matches well with the intermittency of solar or wind-generated renewable electricity, and therefore our device can provide clean drinking water without the need for battery energy storage.

### Robustness against Complex Water Chemistry

**Figure 5.**
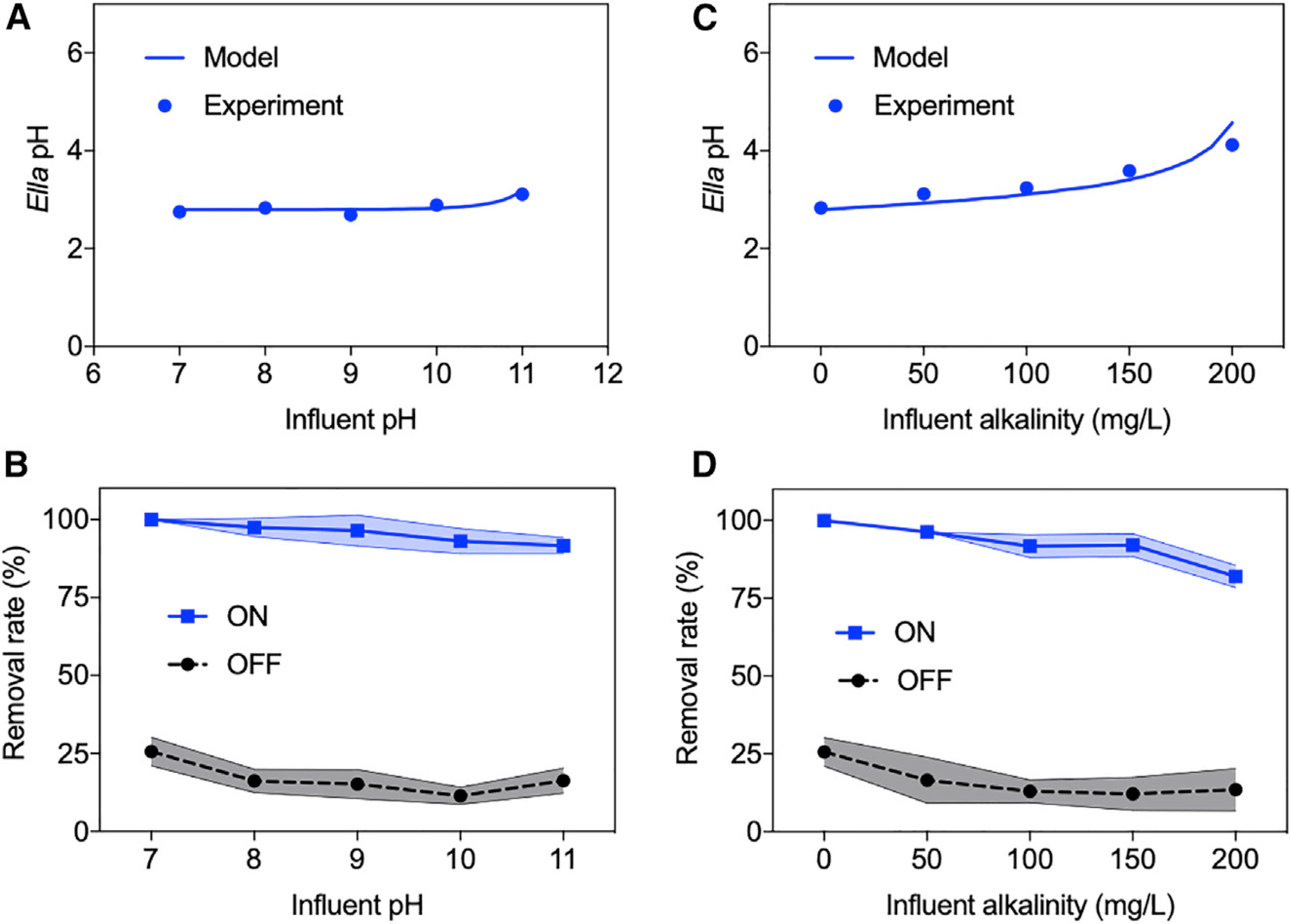
Robustness of Electro-Fenton-like Platform against Complex Water Chemistry (A and C) Acidic pH produced by *Ella* process. (B and D) BPA removals when the electric current was turned off and on. (A and B) Influents were adjusted to different initial pHs, and (C and D) sodium carbonate was added to influents for synthetic alkalinity and influents were maintained at neutral. *Ella* pH denotes the average of solution pHs from 2 middle sampling ports. Shaded areas in (B) and (D) indicate the error range of obtained data.

Complex water environments are typically encountered in water treatment practices, posing challenges to downgradient treatment. For instance, water bodies receiving leachates from industries could be highly basic and require a pH neutralization process such as CO_2_ sequestration before biological/chemical treatment.^38^ In this study, contaminants in a synthetic basic solution are treated by the EFL platform. Influent solutions of pH 7–11 were effectively acidified to ~pH 3 under 100 mA electric current and a 15-mL/min flow rate, in good agreement with modeling results (Figure 5A). Consequently, BPA removals approached 100% under the *Ella* process-mediated acidic environment, regardless of influent pH, whereas <22.4% of BPA was removed when the electric current was turned off (Figure 5B).

The potential of water bodies to neutralize protons, namely water alkalinity, represents another challenge for acid-demanding chemical treatments due to the buffering carbonate ions, expressed as equivalent milligrams of CaCO_3_/L.^39^ Specifically, a substantial amount of acid is required to overcome high water alkalinity for the conventional Fenton process. BPA removal in synthetic solutions containing 0–200 mg CaCO_3_/L water alkalinity was tested by the EFL platform. The results show that the solution of higher alkalinity was less prone to acidification by the *Ella* process, but the acidic zone was still below pH 4.14 in all of the tests under the 100-mA electric current and the 15-mL/min flow rate (Figure 5C). This acidity led to an 84.5%–100% removal efficiency of BPA, which is significantly higher than that when the electric current was turned off (Figure 5D). The acidity produced by the *Ella* process could be tuned against the water buffering capacity by adjusting the applied electric current and hydraulic flow rate. For instance, a combination of 60 mA electric current and 2 mL/min flow rate is able to acidify a solution of 500 mg CaCO_3_/L alkalinity from pH 8 to pH 3.5.^40^

### Test with Field Water Samples

The results demonstrate the effectiveness of the EFL platform in synthetic solutions of complex water chemistry. Furthermore, in this study, water samples extracted in the field from surface or groundwater sources were used as the matrices for BPA removal, with intrinsic conductivity supporting electrochemical water splitting. Water-quality characteristics are shown in Table S4. Treated water bodies were acidified to pH 2.9–4.04 by the *Ella* process, and 91.7%–97.2% BPA removals were achieved by the mediated Fenton-like process (Figure S17).

### Techno-economic Analysis

Compared with the conventional Fenton process, the required energy for electrochemical water splitting by this integral EFL platform is estimated to be 1.39 kWh/m^3^ influent, which translates into a cost of $0.091/m^3^ based on the average US industrial electricity rate ($0.0653/kWh).^41^ This value is subjected to optimization based on applied electric current, hydraulic flow rate, and solution conductivity, but it only constitutes ~10% of unit operating costs by modern water treatment plants.^42^ The cost of γ-Al_2_O_3_ and FeCl_3_•6H_2_O is estimated at $0.5/kg^43^ and $0.3/kg,^44^ respectively. This means it takes <$0.0005 to synthesize 1 g FeOCl/Al_2_O_3_ catalyst, and we used ~50 g of synthesized catalyst particles for our device. By considering the long-term stability of this catalyst, its cost is indeed negligible. Furthermore, *in situ* electrochemical synthesis of highly concentrated H_2_O_2_^45,46^ could be used as a replacement for externally supplied H_2_O_2_ in the future, eliminating the chemical cost.

Conventional Fenton reactions produce a tremendous amount of iron sludge, which needs treatment and induces huge costs. The cost of sludge disposal post-water treatment is ~$40–$100/ton.^16,52^ However, the total cost is unclear since different amounts of iron are used for different bodies of water. Nevertheless, it has been reported that the cost for treating sludge is 10%–50% of the total cost, and sometimes can be up to 60%.^17^ Therefore, we believe the overall cost of our system is lower than conventional Fenton reactions. Our design avoids the iron sludge generation, which not only reduces the cost but also is more environmentally friendly. In general, however, the techno-economic analysis here is quite preliminary and will require more in-depth work in the future.

Moreover, we would like to mention future directions of scaling up the device. First, the catalyst should be cheap and easy to produce. One can certainly replace FeOCl with other Fenton catalysts, such as the nano MnO_2_ catalyst,^53,54^ since our system is not solely dependent on the FeOCl. Second, to ensure sufficient contact time between H_2_O_2_ and catalyst, the two electrodes should be distant enough. An outcome of this design strategy is the high voltage applied onto the two electrodes to drive the migration of proton and other ions. This will eventually increase the cost in electricity, and hence there exists a “sweet spot” where electricity cost and catalysis performance reach a trade-off balance. Finally, the immobilization of the FeOCl catalyst should be adapted for industrial-scale operation. For example, it has been widely reported that catalyst nanoparticles could be ground into large particles via ball milling.^55–57^ This scalable method leads to very stable catalyst immobilization.

The EFL water treatment platform developed in this study shows the potential for practical implementation by the water treatment industry after upscaling. The fast response of the EFL platform in removing organic pollutants especially allows for smart controls at low cost. In addition, it possesses the easy-to-(un)install feature that makes it affordable and compatible with other connecting units of a centralized water treatment facility. For instance, a feasible niche of this device is to function as an electrochemical filter before advanced purification units requiring the removal of fouling hazards such as organic molecules and biofilm-forming bacteria.^47,48^ On the small size end, our EFL platform can be redesigned to provide clean drinking water for individual families without using the electrical grid, by coupling with cheap photovoltaic sources.

Here in this work, an EFL platform wherein an electrochemically produced acidic environment supports the heterogeneous Fenton-like reaction was developed for a high-throughput water treatment purpose. This integrated platform relies on an electrolytically localized acid compartment, referred to as the *Ella* process. The acidic pH produced by the *Ella* process (100 mA electric current and 15 mL/min flow rate) mediated a 10.8-fold increase in HO^•^ yield, which contributes to the complete removal of contaminants in the effluent. The EFL platform shows strong stability for long-term use, tolerance of high water flux, and effectiveness against complex water chemistry for organic contaminant removal. The proposed setup configuration is advantageous in that it provides a high degree of automation that enables water treatment by controlling the electric power, and also in the low operating cost that most modern water treatment plants strive for, both of which are especially attractive for industrial applications.

## EXPERIMENTAL PROCEDURES

### Resource Availability

#### Lead Contact

Requests for further information and resources and reagents can be directed to the Lead Contact, Prof. Ju Li (liju@mit.edu)

#### Materials Availability

This study did not generate new unique reagents.

#### Data and Code Availability

The authors declare that the data supporting the findings of this study are available within the article and the Supplemental Information. All other data are available from the Lead Contact upon reasonable request.

#### Chemicals

FeCl_3_•6H_2_O (Honeywell Fluka) was used to synthesize the FeOCl nanocatalyst, and H_2_O_2_ was purchased from Fisher Scientific. γ-Al_2_O_3_ (Alfa Aesar) was used as a support to immobilize FeOCl. BPA (Sigma-Aldrich), ibuprofen (Alfa Aesar), atrazine (Sigma-Aldrich), carbamazepine (Sigma-Aldrich), 4-chlorophenol (Acros Organics), 4-nitrophenol (Acros Organics), rhodamine B (Harleco), reactive blue 19 (Sigma-Aldrich), and orange II (Acros Organics) were used as substrates for the Fenton-like process treatment. Other chemicals (i.e., sodium sulfate [Na_2_SO_4_] as the supporting electrolyte, sodium carbonate [Na_2_CO_3_] for synthetic alkaline water, ethanol as a HO^•^ radical scavenger, and methanol and acetonitrile as the mobile phase for high-performance liquid chromatography [HPLC]) were purchased from Fisher Scientific. Pure water was used throughout the work, except in the field water studies.

#### Synthesis of FeOCl

A total of 2 g ground FeCl_3_•6H_2_O powder was put at the bottom of a ceramic crucible and then tightly sealed with aluminum foil. The crucible was heated at an 8°C/min rate up to 200°C and maintained for 2 h in a muffle furnace. After heating, the crucible was naturally cooled down to room temperature. The cooling process took ~1 h. The formed dark red FeOCl plates were ground into fine powder and then washed with ethanol at least three times until the eluent was colorless to remove residual FeCl_3_ impurity. Eventually, the synthesized and purified FeOCl was stored in a dry 15-mL Corning tube, and then put in a chemical fume hood under constant ventilation drying.

#### Synthesis of FeOCl/Al_2_O_3_

A total of 10 g ground γ-Al_2_O_3_ microparticles were mixed with 5.97 g FeCl_3_•6H_2_O (i.e., theoretical 10 wt% Fe loading) under vigorous vortex for 10 min until the powder mixture turned homogeneously yellow. The powder was then transferred into 5-mL glass tubes with air-tight caps and further sealed with Teflon bands to prevent the vaporization of crystalline water in FeCl_3_•6H_2_O. The glass tubes were heated under 80°C for 12 h, during which FeCl_3_•6H_2_O (melting point 37°C) infiltrated the γ-Al_2_O_3_ pores. The obtained powder was transferred into crucibles for the synthesis of FeOCl/Al_2_O_3_ using the same conditions as FeOCl synthesis. Produced FeOCl/Al_2_O_3_ was extensively washed with ethanol before use.

#### Characterization of Materials

The crystalline phase of samples was measured using a D/MAX-2200 XRD analyzer equipped with a rotation anode using CuKα radiation (λ = 0.1541 nm). FESEM photography was performed on an S-4800 instrument at an accelerating voltage of 15 kV. High-resolution transmission electron microscopy (HRTEM) images were obtained on a JEOL JEM-2100F transmission electron microscope at an accelerating voltage of 200 kV. The chemical composition and the binding states on the surface of the FeOCl specimen were carried out on a Thermo Scientific ESCALAB 250 Xi XPS microprobe with monochromatic X-ray (Al Kα, 1,486.6 eV) radiation as an excitation source. The measurements of specific surface area, pore volume, and pore size distribution in the 2–500 nm range were carried out using a Micromeritics ASAP 2020 instrument by nitrogen adsorption/desorption at 77.3 K (−194.85°C).

#### Flow-through Column Reaction

The experimental column (15 × 5 cm OD, 0.32 cm wall thickness) is made with cast acrylic tubes and Teflon rods with O-rings as the top and bottom caps. Mixed metal oxide sintered titanium (Ti/MMO, IrO2:RuO2 50:50 mixture deposited on titanium substrate) mesh electrodes were inserted parallel to one another at a distance of 9 cm. Titanium hex nuts and threaded rods were used to connect Ti/MMO electrodes with the electric power source. Gum rubber was sealed on the external titanium rod to prevent contact with both electrodes. Four sampling ports using tube adaptors (0.79375 cm tube to 0.3175 cm National Pipe Tapered [NPT] male) were installed at an equal distance (3 cm) between the electrodes.

To load catalysts into the reactor, the FeOCl/Al_2_O_3_ catalyst (~50 g) was filled between the cathode and anode, and rest spaces were filled with fine silica sand particles. The silica sand particles were extensively washed with pure water and then oven dried at 80°C. Filter papers were placed at the bottom and top of the sand zones to prevent particles from draining into the effluent.

Initially, a neutral solution (pH 7) of 10 μM BPA was used to condition the column until the effluent BPA concentration matched the influent to reach adsorption equilibrium. After BPA breakthrough, another neutral solution containing 10 mM H_2_O_2_, 10 μM BPA, and 5 mM Na_2_SO_4_ electrolyte was pumped up by a peristaltic pump into the vertically aligned column at a rate of 15 mL/min, with a 100-mA electric current applied to the electrodes. A steady-state acid compartment was formed between electrodes after 60 min, and BPA removal was tested. One-milliliter solutions sampled from each port and influent/effluent were neutralized with 1 mL 20 mM phosphate buffer (pH 7) to avoid following analysis inaccuracy due to pH inconsistence. Samples after filtration with the 0.45 μm polyvinylidene fluoride (PVDF) membrane (13 mm diameter, Jin Teng) were measured by HPLC (Agilent 1200 Infinity Series) equipped with an Agilent Eclipse AAA C18 column (4.6 × 150 mm). BPA was separated by 0.5 mL/min methanol/water 60/40 mobile phase and detected at 228 nm wavelength using the Agilent 1260 diode array detector. The degradation of other substrates was also tested, and quantification methods are indicated in Table S3.

In certain assays, the solution pH, flow rate, and synthetic alkalinity (i.e., 1 mM Na_2_CO_3_ is equivalent to 100 mg CaCO_3_/L water alkalinity) were subjected to changes, as indicated, while other parameters were fixed, as stated above. For the electricity responsiveness test, an experiment was initiated by turning on the 100-mA electric current after conditioning the column, without reaching steady state. For BPA removal in field water bodies, water samples 1 and 2 were taken from lakes in Boston, Massachusetts, and water samples 3 and 4 were taken from underground sources of Superfund sites in Puerto Rico. They were stored in a 4°C cold room. Characterizations of the field water samples are shown in Table S4. Water samples were filtrated through 0.45 μm PVDF membranes (47 mm diameter, EMD Millipore) before use to remove suspended particles. Solution pH was not adjusted after the addition of H_2_O_2_ and BPA, and solution intrinsic conductivity supported electrochemical water splitting. BPA concentrations in the influent and effluent were analyzed during steady state.

#### Hydroxyl Radical Quantification Method

The total HO^•^ radical amount was quantified via a reported benzoic acid oxidation method.^49^ Briefly, the accumulated HO^•^ radical amount equals that of generated *p*-hydroxybenzoic acid, a product of HO^•^ radical and benzoic acid reaction, multiplied by a conversion factor of 5.87. In the batch reaction, 100 mL solutions of 5 mM benzoic acid, 10 mM H_2_O_2_, and 0.2 g/L catalyst (i.e., free FeOCl or immobilized FeOCl/Al_2_O_3_) were stirred for 30 min under the indicated solution pH. In the flow-through reaction, after conditioning the column with 5 mM benzoic acid until breakthrough, influent solutions containing 5 mM benzoic acid, 10 mM H_2_O_2_, and 5 mM Na_2_SO_4_ at neutral pH passed through the column at a rate of 15 mL/min, with or without the 100-mA electric current applied to the electrodes. Effluent samples were collected during steady state. The concentration of generated *p*-hydroxybenzoic acid was analyzed by HPLC with the mobile phase of 0.5 mL/min methanol/water 20/80 and the detection wavelength at 255 nm using the same column for BPA analysis.

#### DFT Calculation Method

##### Geometry Optimization.

DFT calculations were performed with PBE (Perdew-Burke-Ernzerhof) functional by using CASTEP^50,51^ as incorporated in Materials Studio 7.0. The ultrasoft pseudopotential (USPP) was used to represent the core-valence electron interaction. The plane wave expansion basis sets with a cutoff energy of 300 eV was used. The k-point sampling of 4 × 4 × 2 within the Monkhorst-Pack special k-point scheme in the Brillouin zone was considered for geometry optimization and energy calculation.

##### Transition State Calculation.

To investigate the pathways of the Fenton process, linear synchronous transit/quadratic synchronous transit (LST/QST) by using the DFT+*U* technique were performed, and the *U* values of O 2p and Fe 3d are 6.3 and 3.0 eV, respectively. The FeOCl (100) surface was considered a reactive surface with 2 fixed atomic layers,^40^ and 2 × 2 × 1 k-point mesh was used. Spin polarization was considered for all of the calculations.

## Supplementary Material

Supplemental Information

Video S1

## HIGHLIGHTS

Electrochemical water splitting under flow-through conditions produces high acidity

This acidity boosts the Fenton-like catalytic activity for organic compound removal

Acidity is verified by modeling and experiment

Stability and robustness of this platform are demonstrated
