# Supplementary material for: A Robust Flow-Through Platform for Organic Contaminant Removal
Source: Cell Rep Phys Sci. Author manuscript; Available in PMC 2021 Aug 5. (PMC8341378; doi:10.1016/j.xcrp.2020.100296)
Supplement: Supplemental Information [file NIHMS1689510-supplement-Supplemental_Information.pdf]

Cell Reports Physical Science, Volume *2*

Supplemental Information

A Robust Flow-Through Platform for Organic Contaminant Removal

Long Chen, Akram N. Alshawabkeh, Shayan Hojabri, Meng Sun, Guiyin Xu, and Ju Li

Supplemental Experimental Procedures

Reactive transport model to estimate the steady-state pH in the *Ella* process

Water splitting is considered as the dominant electrolysis reaction in this system. This process will produce an acid front after the anode and gets neutralized after passing through the cathode.

$$\text{Anode: } 2H_2O \rightarrow O_2 + 4H^+ + 2e^- \quad (1)$$

$$\text{Cathode: } 2H_2O + 2\,e^- \rightarrow H_2 + 2OH^- \quad (2)$$

Competing reactions may exist depending on the electrolyte composition and concentration which reduces the current efficiency of the system. However, in this study we assume 100% efficiency for water electrolysis on both electrode surfaces.

Transport process in the column is expressed by Nernst-Plank's equation in dilute electrolytes.

$$J_i = uC_i - D_{H_\mathrm{i}} \nabla C_i - u_i z_i F C_i \nabla \phi \quad (3)$$

Where u is the pore water velocity and J_i, C_i, D_{H_i}, u_i, and z_i are the total flux, concentration, hydrodynamic dispersion coefficient, mobility, and the charge of ith species, respectively. Species mobility, u_i, can be estimated by Einstein relation.

$$u_i = \frac{z_i F D_i}{RT} \quad (4)$$

Hydrodynamic dispersion, D_H, is a function of the pore water velocity and is represented as $D_H = \tau D_0 + \alpha_L u$, where the tortuosity, τ, is a measure of effective transport path through media, D_0, is the molecular diffusion coefficient, and α_L, is the longitudinal

dispersivity. Many studies are conducted to evaluate the hydrodynamic dispersion coefficient[1]. This factor in large scale systems is estimated by column tracer test or fitted models of similar experiment characteristics.

In systems with supporting electrolyte, migration term can be neglected. Therefore, the governing equation for a transient simulation is obtained by conservation of mass.

$$\frac{\partial c_i}{\partial t} = -\nabla J_i + R_i \tag{5}$$

Where R_i is the reaction rate of species i in the bulk fluid. Electric field in the electrolyte can be calculated considering conservation of charge in the electrolyte, knowing that current arises from the motion of all charged species.

$$i = -\kappa \nabla\phi - \kappa F \sum_i z_i D_i \nabla C_i \tag{6}$$

Where the first term is ohmic potential and the second term is concentration-caused potential. In systems with supporting electrolyte, the concentration gradient can be neglected and the equation reduces to

$$i = -\kappa \nabla\phi \tag{7}$$

Where

$$\kappa = F^2 \sum_i z_i^2 u_i C_i \tag{8}$$

The inlet boundary condition is considered Dirichlet boundary with a constant feed concentration over time.

$$C(x = 0, t) = C_0 \tag{9}$$

The outlet boundary condition is a Neumann boundary condition:

$$\nabla C_i = 0 \tag{10}$$

Fluxes on the surface of the electrodes are shown as followed.

$$J_i|_{x=electrode+\partial x} = J_i|_{x=electrode-\partial x} + J_{eRXN,i} \tag{11}$$

where, $J_{eRXN,i}$, is the inward electrochemical source/sink flux for i^{th} species and is calculated in a constant current operating condition using Faraday's law.

$$J_{eRXN,i} = \frac{I}{z_i F A} \tag{12}$$

I applied current

z_i number of electrons participating for each mole of species i produced/consumed

F Faraday's constant, $96485\ c/eq$

A Electrode surface area

The homogeneous phase reactions included in the model are assumed to reach equilibrium.

$$\sum_{j=1}^{N_c} \upsilon_{ij} C_j = 0 \qquad \text{for } i = 1, \dots, N_x \tag{13}$$

N_c total number of species

N_x total number of reactions

υ_{ij} stochiometric constant for j^{th} species in the ith reaction

There have been many studies on integration of species transport and equilibrium reaction models[2-6]. In this study, reaction simulations and species concentrations are determined by PHREEQC computer program[7]. PHREEQC is a computer program which

is designed and optimized for aqueous geochemical reactions with a strong database. Nardi et al. have developed a comprehensive geochemical model using COMSOL Multiphysics for transport calculations and PHREEQC for geochemical reaction calculations[8]. Paz Garcia et al.[5] have obtained a similar approach in simulation of electrochemical remediation reactors using COMSOL Multiphysics with an interface with PHREEQC. In our study, we used MATLAB as the main platform for numerical calculation with an interface with PHREEQC for chemical reaction calculations. The interface written in MATLAB is used to transfer data between MATLAB and PHREEQC using Microsoft COM (Component Object Model). *Phreeqc.dat* is the thermodynamic database used in this study for equilibrium calculations. The reactions in the bulk fluid include acid/base reactions, water association/dissociation reaction, and redox reactions. Due to the characteristics of the aqueous reactions in this system, local equilibrium is considered.

In this study, hybrid Finite Volume differencing scheme is used for spatial discretization and implicit scheme is used for temporal discretization with a non-iterative sequential solver for integration of reactions in the simulation. This scheme guarantees continuity of the concerned variables and stability of numerical simulation. The hybrid scheme will evaluate the differencing scheme based on local Peclet number.

$$Pe = \frac{u}{D/L} \tag{14}$$

If the local Peclet number is small, diffusion is dominant and central differencing scheme results in smaller error. However, if the advection is dominant, upwind scheme is used to more accurately capture the impact of advection[9].

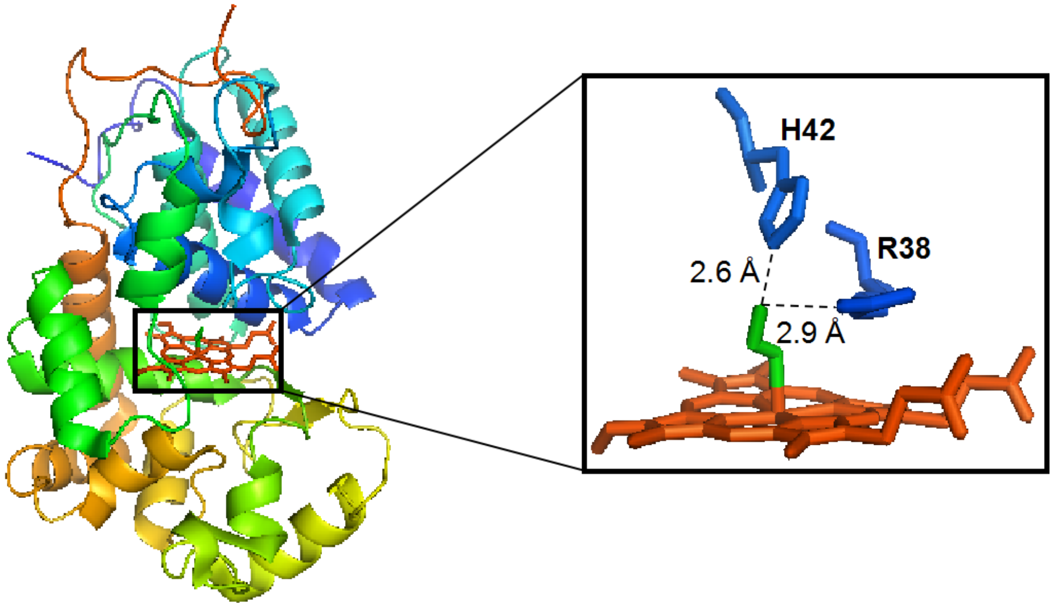

Figure S1. Horseradish peroxidase (HRP) protein structure (PDB entry: 1W4W). Right panel: formate as an allosteric inhibitor binds to the iron atom of heme compound, and this complex is stabilized by H42 and R38 of HRP peptide.

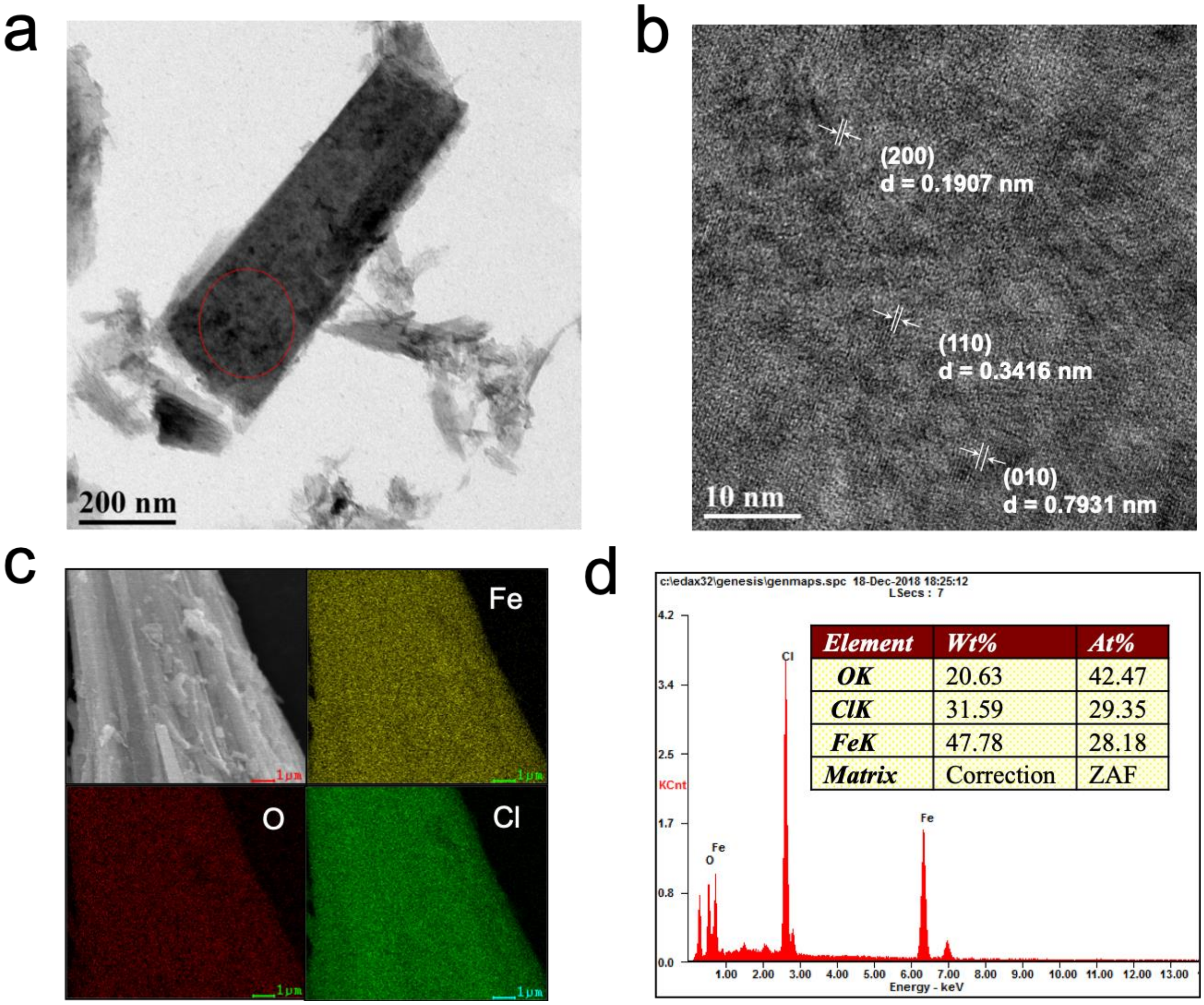

Figure S2. Characterizations of synthesized FeOCl nanosheet catalyst. (**a**) TEM, (**b**) HRTEM, (**c**) element mapping and (**d**) element ratio measurement from EDX analysis.

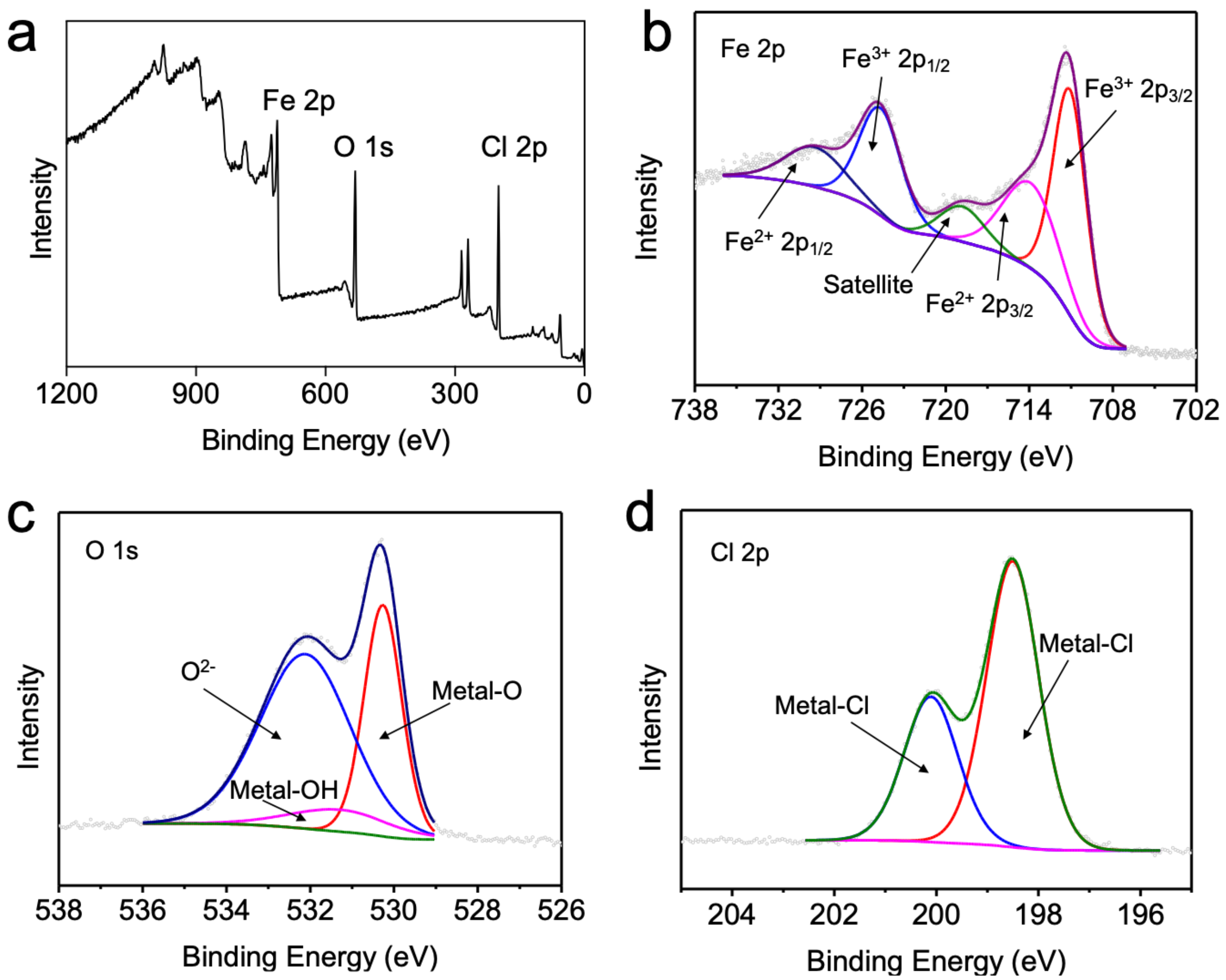

Figure S3. XPS characterization of FeOCl sample. (**a**) Survey spectrum, (**b**) Fe 2p, (**c**) O 1s, and (**d**) Cl 2p.

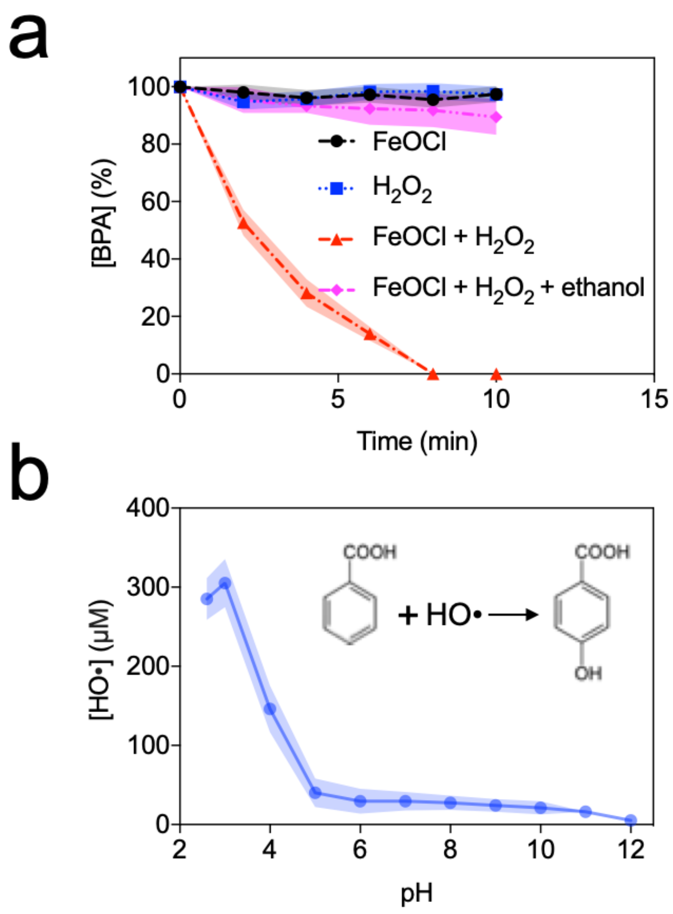

Figure S4. Activity of synthesized FeOCl. (**a**). BPA degradation by FeOCl/H_2O_2 reaction. (**b**). Quantification of generated $HO^{\bullet}$ radical by FeOCl/H_2O_2 reaction at different pH.

Note S1. The activity of synthesized FeOCl sample was tested by decomposing H_2O_2 for BPA removal. It was shown that 0.2 g/L FeOCl with 10 mM H_2O_2 at pH 3 could remove 86% 10 μM BPA in 6 min and 100% BPA in 8 min. The role of $HO^{\bullet}$ radical in BPA removal was verified by ethanol as a scavenger, since it fully inhibited BPA removal via quenching $HO^{\bullet}$ radical. The catalytic activities of FeOCl at different pHs were compared. Results showed that, the optimum pH for FeOCl is around pH 3 as it mediated the highest yield of $HO^{\bullet}$ radical.

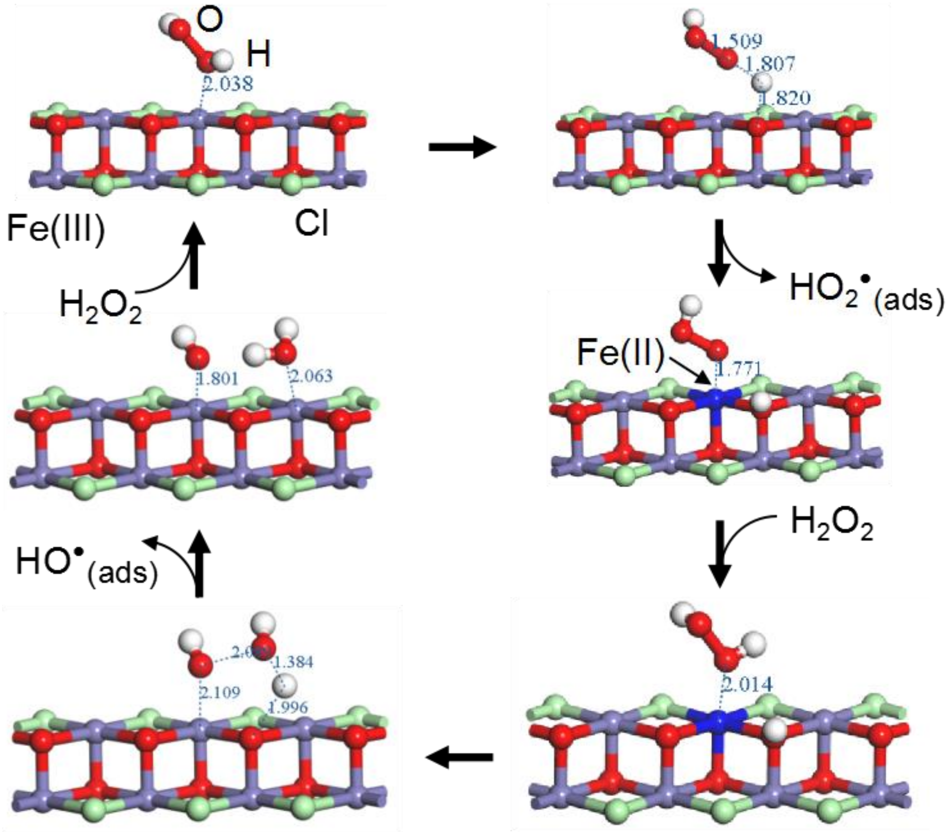

Figure S5. Peroxidase-like catalysis mechanism of FeOCl/H_2O_2 reaction.

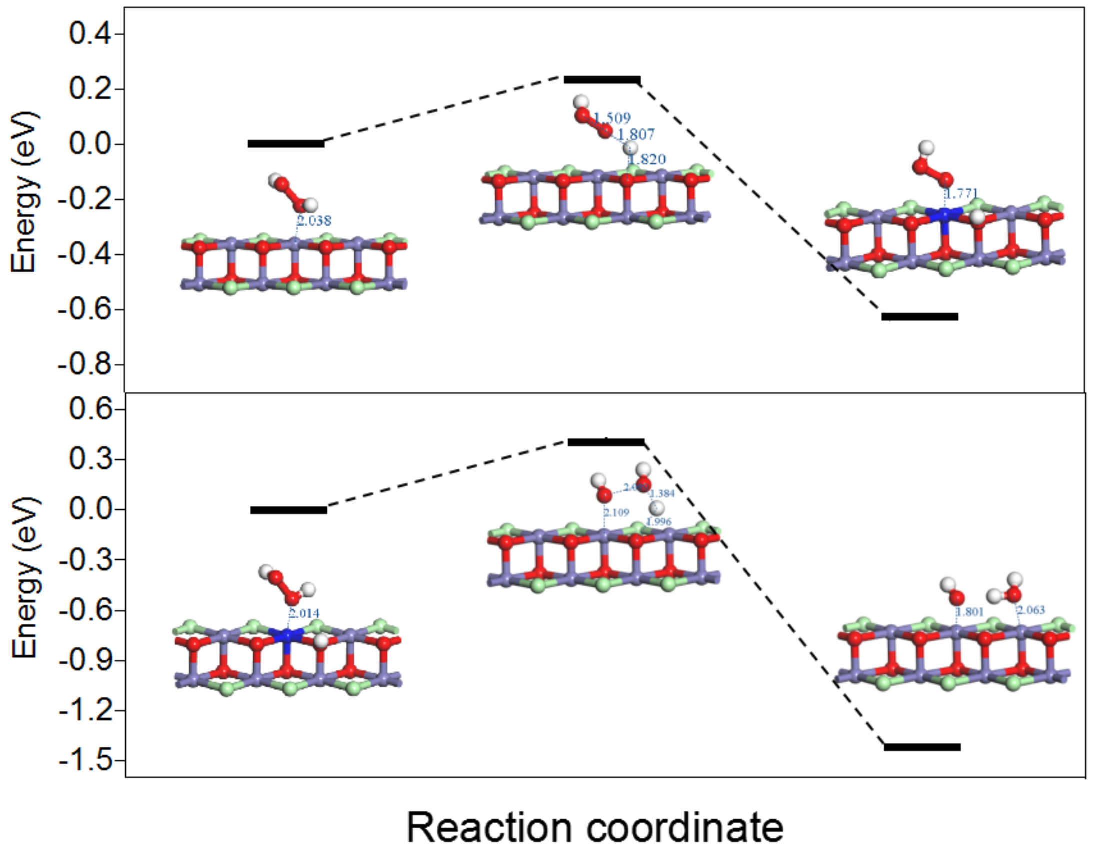

Figure S6. Reaction energy in step 1 (top) and step 2 (bottom) based on DFT calculation. Step 1: $Fe^{III}OCl$ is at first reduced by H_2O_2 into $Fe^{II}OCl$, and Step 2: the derived $Fe^{II}OCl$ then decomposes H_2O_2 into $HO^{\bullet}$ radical via homolytic cleavage.

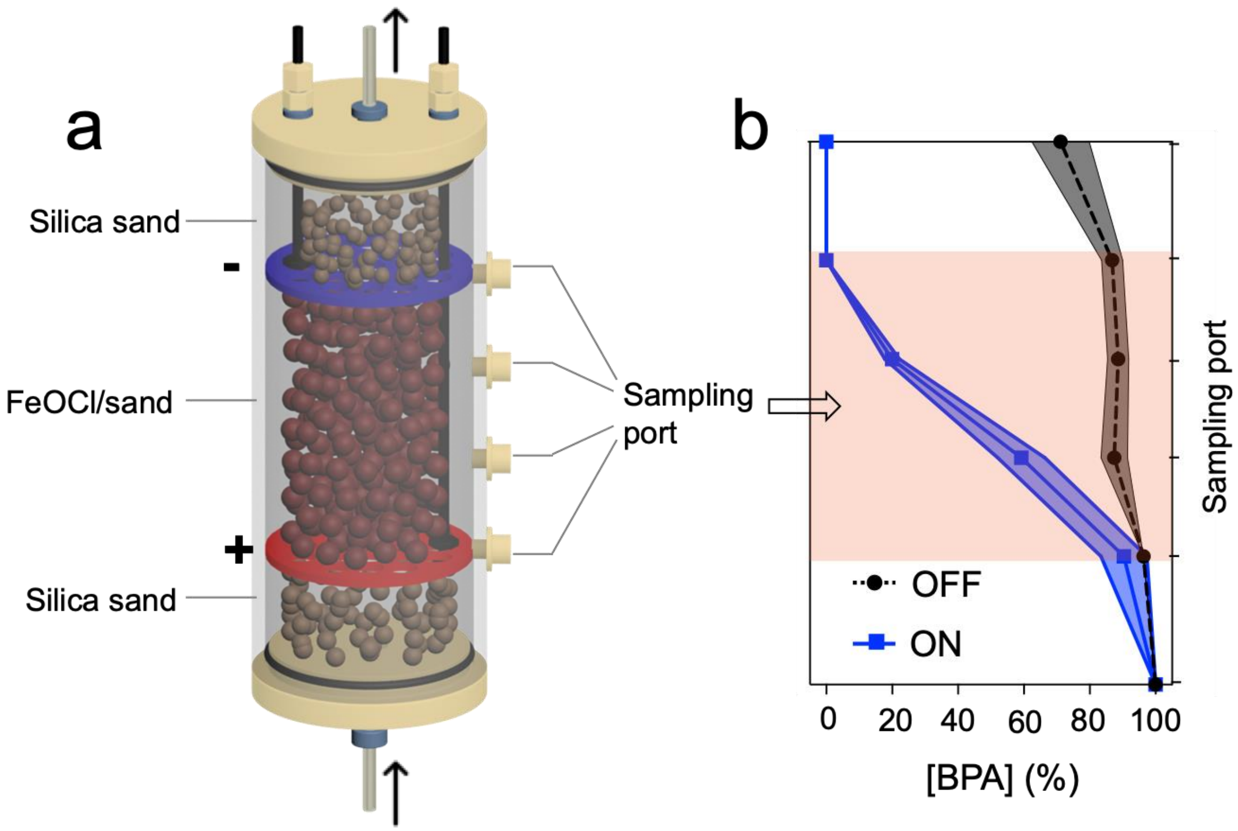

Figure S7. BPA removal by EFL platform loading unimmobilized FeOCl. (**a**). Illustration of reactor components, (**b**). BPA removals from each sampling port as the electric current was turned on and off.

Note S2. 5 g FeOCl powder was mixed well with 50 g sand particles under vortex for 20 min, and the mixture was then filled in between the cathode and anode, and all other spaces were filled with sand particles to reduce hydrodynamic perturbation. A neutral solution containing 10 mM H_2O_2, 10 μM BPA, and 5 mM Na_2SO_4 as electrolyte was pumped up into the vertically aligned reactor at a rate of 15 mL/min. A steady-state acidic-compartment was formed between the cathode and anode under a current of 100 mA after 60 min, and BPA removals at each sampling port was tested.

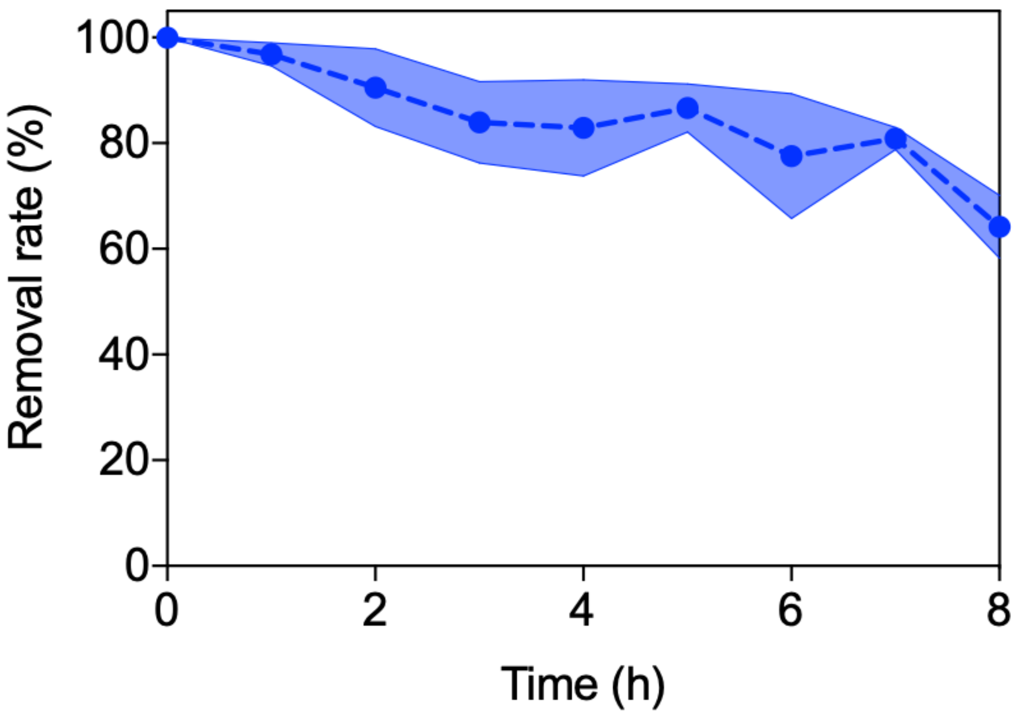

Figure S8. Stability test of BPA removal by reactor loading unimmobilized FeOCl (shown in **Fig. S7a**) for 8 h.

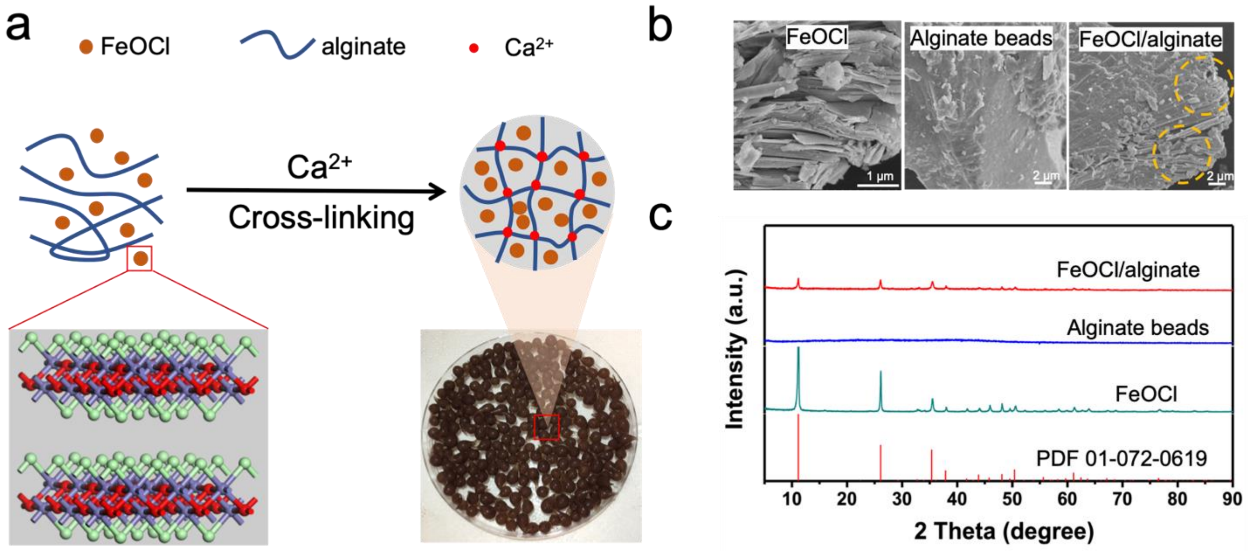

Figure S9. Immobilization of FeOCl on alginate hydrogel. (a) Illustration of immobilization strategy, (**b**) SEM and (**c**) XRD characterizations.

Note S3. 1.5 g synthesized FeOCl and 3 g alginate were at first sufficiently mixed via vigorous vortex, and then 100 mL DI water was poured into the beaker containing well-mixed FeOCl and alginate. A clean glass stick was used to stir the solution until it turned into homogeneous red paste. A syringe was then used to drop the red slurry into 0.1 M $CaCl_2$ solution, and formed FeOCl/alginate beads were allowed to harden for 2 h. The as-prepared FeOCl/alginate beads were then washed with DI water and loaded into the reactor for subsequent experiments.

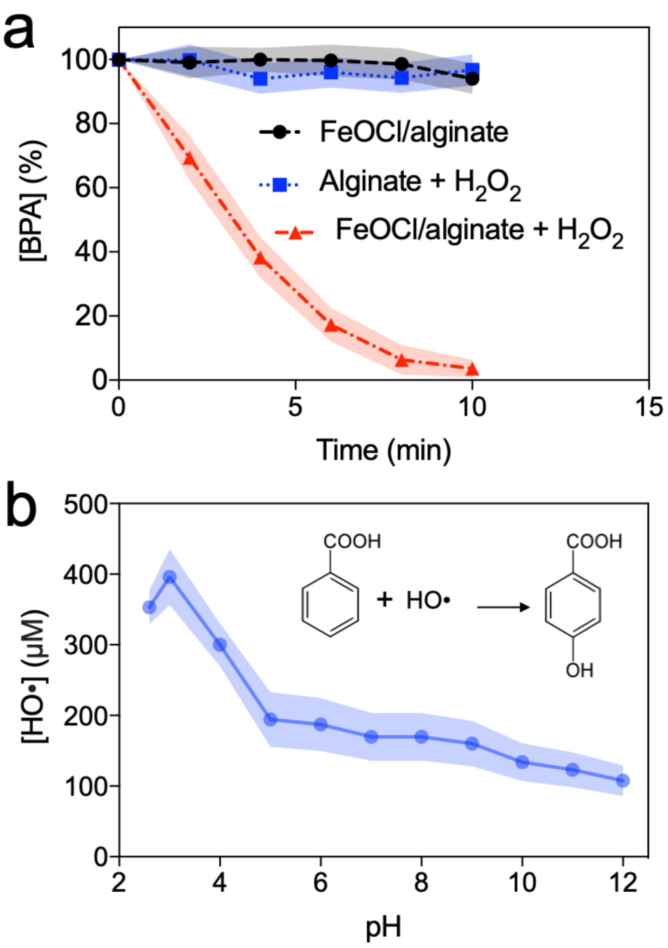

Figure S10. Activity of synthesized FeOCl/alginate. (**a**). BPA degradation by FeOCl/alginate and H_2O_2 reaction. (**b**). Quantification of generated HO$^\bullet$ radical by FeOCl/alginate and H_2O_2 reaction at different pH. Reaction conditions: 0.075 g/mL FeOCl/alginate (dry weight), 10 mM H_2O_2. For (**a**) 10 µM BPA as substrate at pH 3, (**b**) 5 mM benzoic acid as HO$^\bullet$ radical trapping agent at different solution pH. HO$^\bullet$ radical was quantified after reaction for 30 min.

Note S4. At pH 3, 0.2 g/L FeOCl and 10 mM H_2O_2 mediated formation of 305.3 µM HO$^\bullet$ radical (**Fig. S4b**) after 30 min, while 0.075 g/mL FeOCl/alginate (dry weight, 1:2 FeOCl:alginate) produced 396.2 µM HO$^\bullet$ radical (**Fig. S10b**). As a result, the specific activity of unimmobilized and immobilized FeOCl was calculated as 1511.5 and 15.8 µM HO$^\bullet$/(g L^{-1}) FeOCl, respectively.

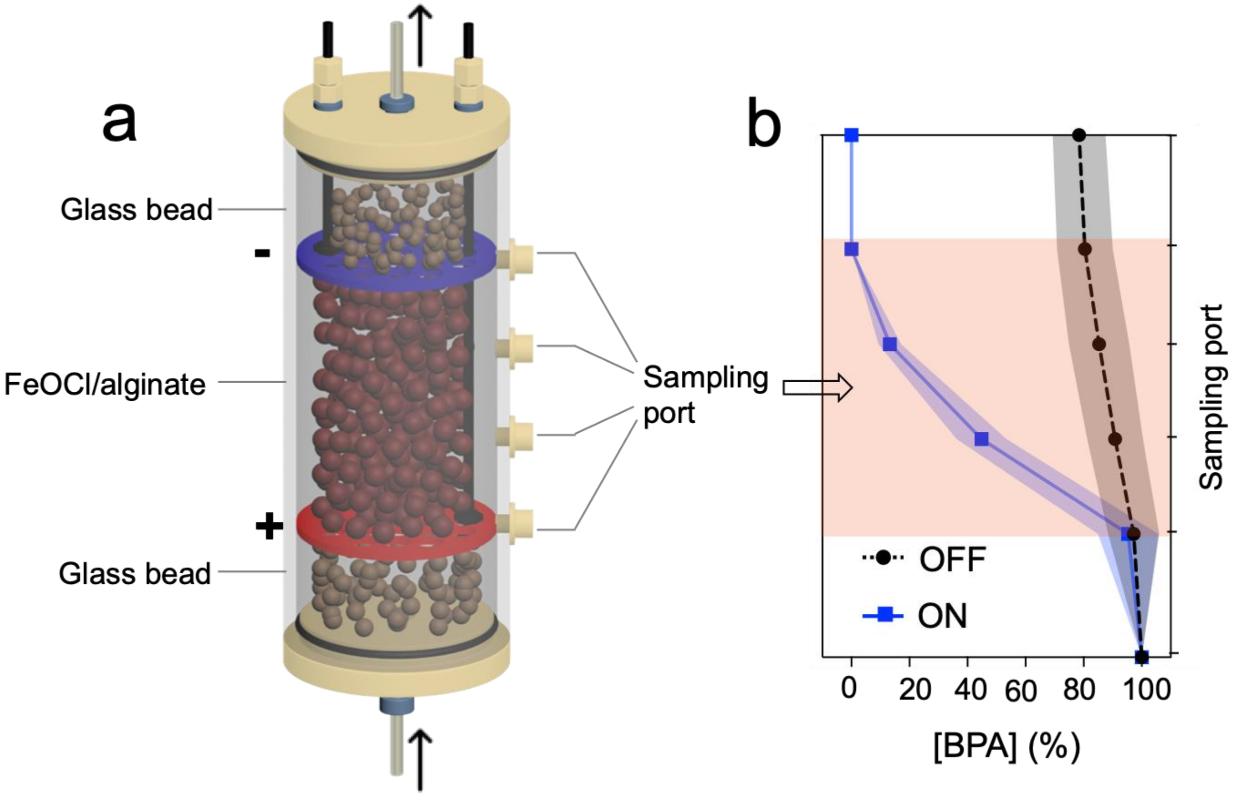

Figure S11. BPA removal by EFL platform loading FeOCl/alginate catalyst composite. (**a**). Illustration of reactor components, (**b**). BPA removals from each sampling port as the electric current was turned on and off.

Note S5. The 4.5 g (dry weight) as-prepared FeOCl/alginate was filled in between the cathode and anode, and all other spaces were filled with glass beads to reduce hydrodynamic perturbation. The pore volume of our reactor between cathode and anode was 60 mL. Typically, a neutral solution containing 10 mM H_2O_2, 10 μM BPA, and 5 mM Na_2SO_4 as electrolyte was pumped up into the vertically aligned reactor at a rate of 3 mL/min. A steady-state acidic-compartment was formed between the cathode and anode under a current of 40 mA after 60 min, and FeOCl/alginate catalyzed H_2O_2 decomposition for BPA removals was tested.

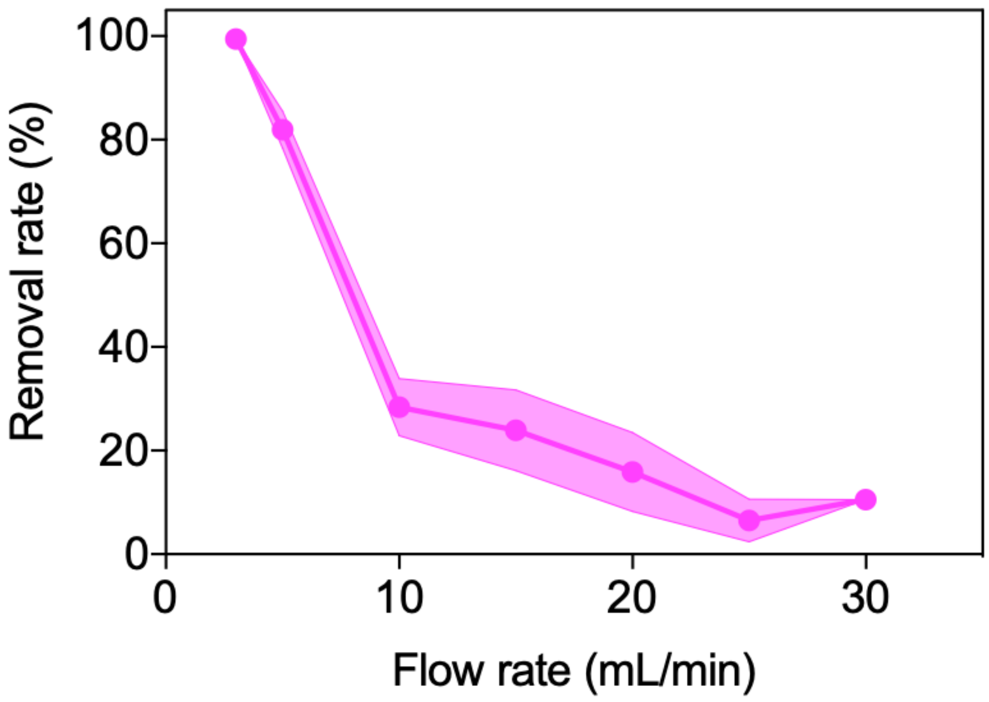

Figure S12. BPA removal under different flow rate by EFL platform loading FeOCl/alginate catalyst composite (as shown in **Fig. S11a**).

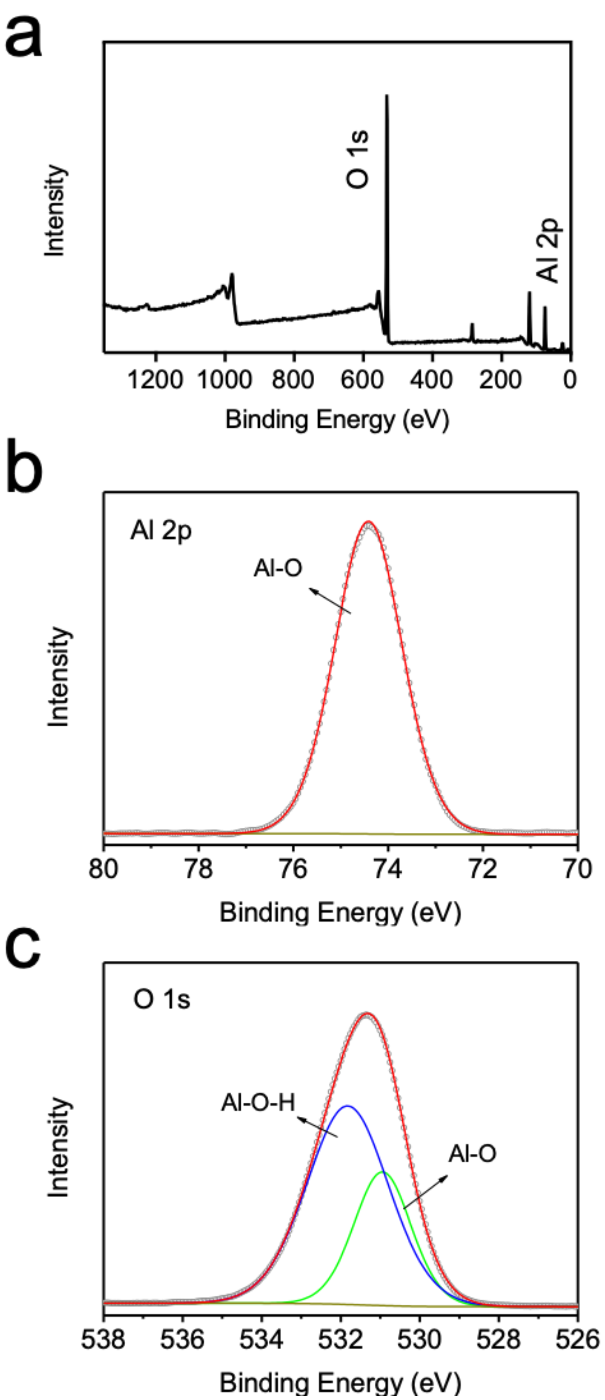

Figure S13. XPS characterization of γ-Al_2O_3. (**a**). Survey spectrum, (**b**). Al 2p, (**c**). O 1s. The observed Al-O-H bond was due to chemisorbed moisture on γ-Al_2O_3 surface.

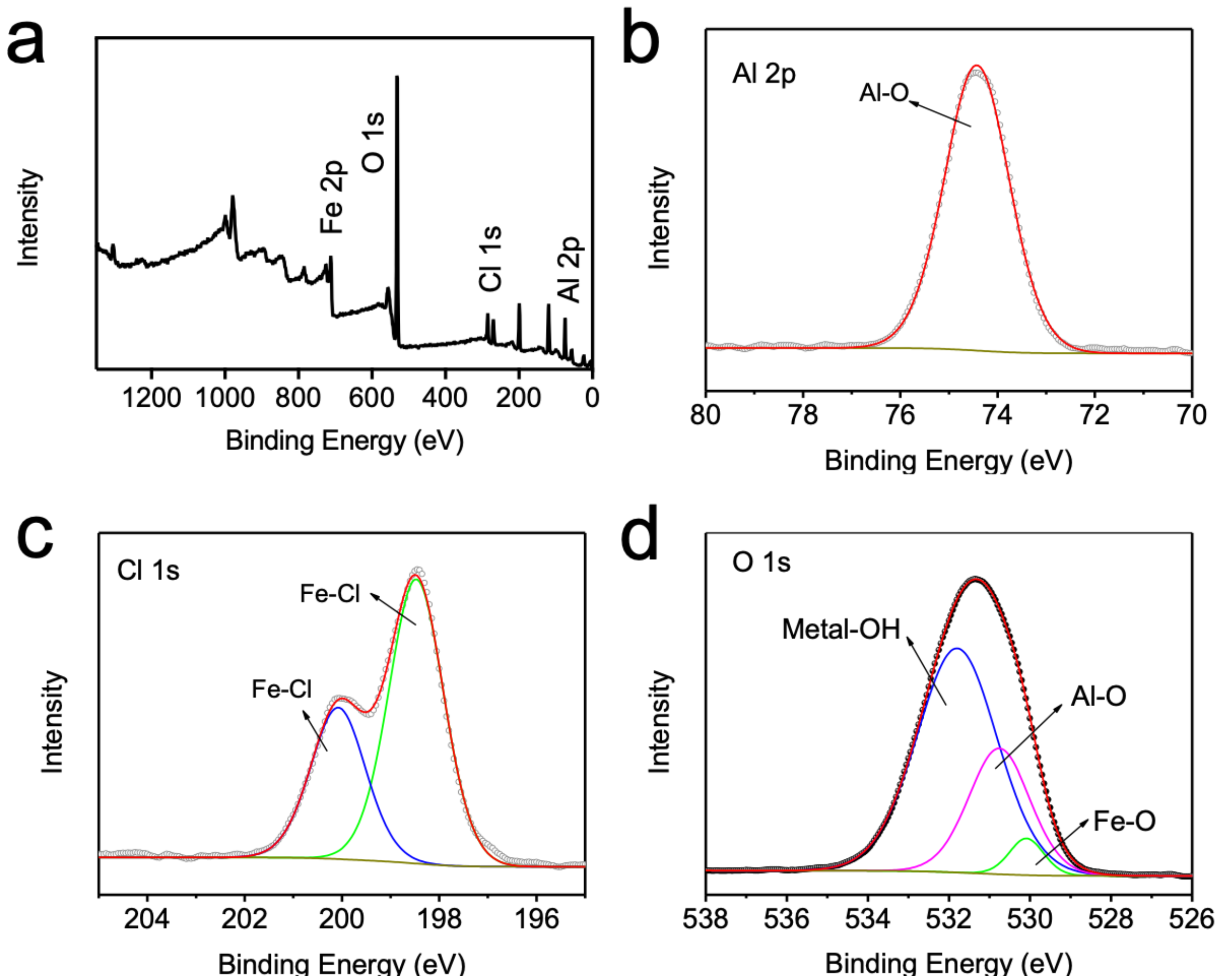

Figure S14. XPS characterization of $FeOCl/Al_2O_3$. (**a**). Survey spectrum, (**b**). Al 2p, (**c**). Cl 1s, (**d**). O 1s. Fe 2p deconvolution spectra are shown in **Figure 2g** in the main text.

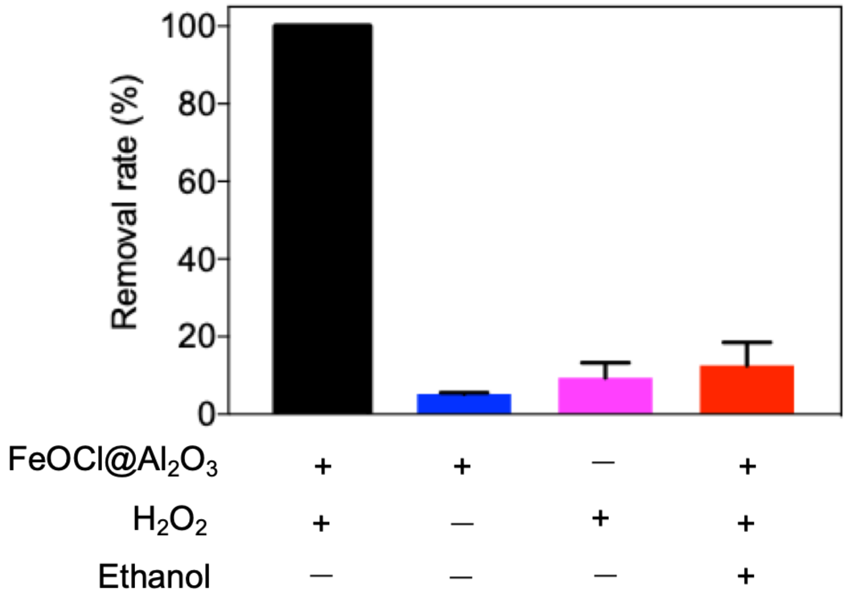

Figure S15. Control experiments of column reaction. BPA removal by the EFL water treatment platform with 1). Both $FeOCl/Al_2O_3$ and H_2O_2, 2). Only $FeOCl/Al_2O_3$, 3). Only H_2O_2, and 4). $FeOCl/Al_2O_3$, H_2O_2, and ethanol. Reaction conditions: 100 mA electric current, 15 mL/min flow rate, 10 mM H_2O_2, 5 mM Na_2SO_4 electrolyte, pH 7. When $FeOCl/Al_2O_3$ was not used, silica sand particles were filled into the catalyst zone instead. 10 mM ethanol was used as $HO^{\bullet}$ radical quencher.

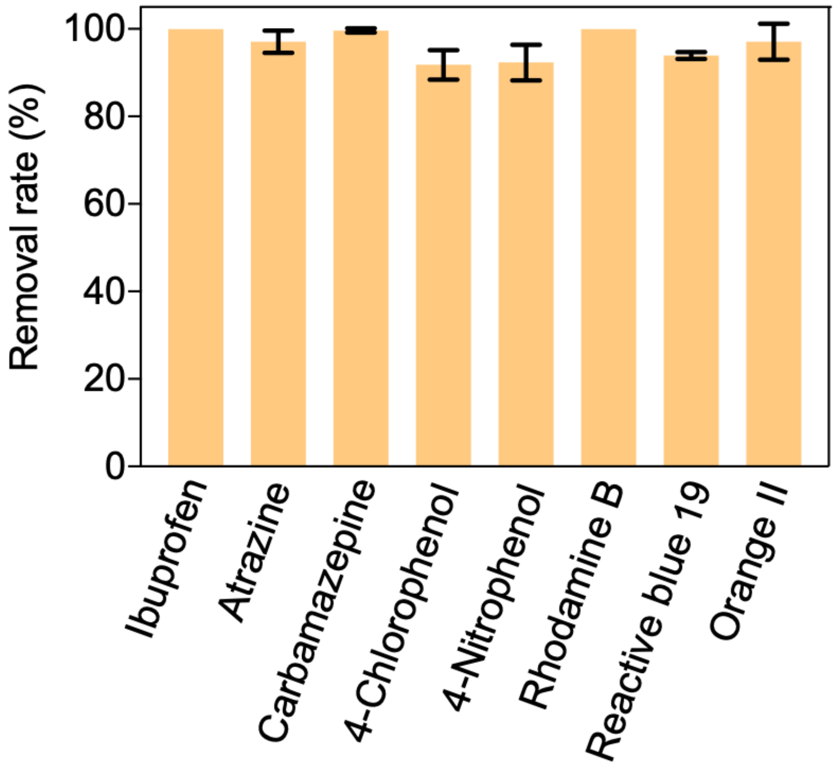

Figure S16. Degradation of recalcitrant organic contaminants by the developed electro-Fenton-like water treatment platform. Initial concentration of these compounds was 10 μM.

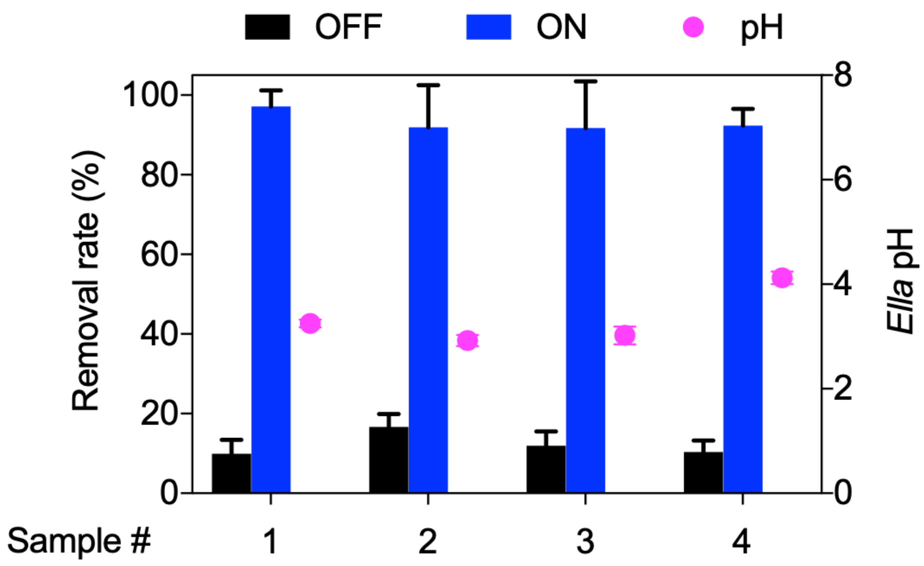

Figure S17. BPA removal by EFL platform in field water matrices. Operating condition: influent (10 mM H_2O_2 and 10 μM BPA in field water sample) was pumped into the column at 15 mL/min, and 100 mA electric current was applied to electrodes. *Ella* pH denotes the average of solution pHs from two middle sampling ports.

Table S1. Summary of heterogeneous Fenton-like catalysts owning peroxidase-mimicking functions in literature.

Catalyst	Activity	Optimum pH	Synthesis method	Reference
Horseradish peroxidase (HRP)	TMB: K_m = 0.434 mM, V_{max} = 10 × 10^{-8} M s^{-1}, k_{cat} = 0.4 × 10^4 s^{-1};<br>H_2O_2: K_m = 3.7 mM, V_{max} = 8.71 × 10^{-8} M s^{-1}, k_{cat} = 0.348 × 10^3 s^{-1};	4	Genetically encoded	10
Fe_3O_4	TMB: K_m = 0.098 mM, k_{cat} = 3.02 × 10^4 s^{-1};<br>H_2O_2: K_m = 154 mM, k_{cat} = 8.58 × 10^4 s^{-1}	3.5	Solvothermal method, co-precipitation method	10-12
$KFePW_{12}O_{40}$	TMB: K_m = 0.346 mM, V_{max} = 3.7 × 10^{-8} M s^{-1};<br>H_2O_2: K_m = 165 mM, V_{max} = 6.9 × 10^{-8} M s^{-1}	4.5	1) Synthesis of $K_3PW_{12}O_{40}$ through hydrothermal treatment of KCl and $H_3[PW_{12}O_{40}]$; 2) iron ion exchange with K^+	13
$H_3PW_{12}O_{40}$	TMB: K_m = 0.11 mM, V_{max} = 43.1 × 10^{-8} M s^{-1};<br>H_2O_2: K_m = 15.89 mM, V_{max} = 42400 × 10^{-8} M s^{-1}	3	N.A.	14
FA-Fe_2SiW_{10}	H_2O_2: K_m = 0.014 mM, V_{max} = 14.24 × 10^{-8} M s^{-1}	4	Co-precipitation method	15
V_2O_5	TMB: K_m = 0.165 mM, V_{max} = 2.4 × 10^{-8} M s^{-1};<br>H_2O_2: K_m = 0.058 mM, V_{max} = 1.4 × 10^{-8} M s^{-1}	4	Hydrothermal method	16
Nanoceria	TMB: K_m = 3.8 mM, V_{max} = 70 × 10^{-8} M s^{-1}	4	Solution reaction at room temperature	17
Co_3O_4	TMB: K_m = 0.037 mM, V_{max} = 6.27 × 10^{-8} M s^{-1};<br>H_2O_2: K_m = 140 mM, V_{max} = 12.1 × 10^{-8} M s^{-1}	6	Heating and precipitation	18
$MnFe_2O_4$	TMB: K_m = 0.112-0.543 mM, V_{max} = (3.53-69.8) × 10^{-4} M s^{-1};<br>H_2O_2: K_m = 0.00146-0.0964 mM, V_{max} = (5.15-71.5) × 10^{-4} M s^{-1}	3.5	Heating and precipitation	19

Ferrocene	TMB: K_m = 0.13 mM, V_{max} = 4.79 × 10^{-9} M s^{-1}, k_{cat} = 4.79 × 10^{-3} s^{-1};<br>H_2O_2: K_m = 50.08 mM, V_{max} = 9.77 × 10^{-9} M s^{-1}, k_{cat} = 9.77 × 10^{-3} s^{-1};	3	Commercially available	20
Ag_3PO_4	TMB: K_m = 0.327 mM, V_{max} = 2.01 × 10^{-8} M s^{-1};<br>H_2O_2: K_m = 0.216 mM, V_{max} = 1.27 × 10^{-8} M s^{-1}	2.5	Solution reaction at room temperature	21
Nanodiamond−gold nanocomposites	OPD: K_m = 6.4-48.7 mM, k_{cat} = 290.4-319.3 mM s^{-1} mg^{-2};<br>H_2O_2: K_m = 89.7-208.7 mM, k_{cat} = 377.6-565.6 mM s^{-1} mg^{-2};	N.A.	Solution reduction and heating	22
MOF-88	TMB: K_m = 0.0796 mM, V_{max} = 3.12 × 10^{-8} M s^{-1};<br>H_2O_2: K_m = 1.06 mM, V_{max} = 1.39 × 10^{-8} M s^{-1}	3	Heating and precipitation	23
Nitrogen-doped graphene quantum dots	TMB: K_m = 11.19 mM, V_{max} = 0.38 × 10^{-8} M s^{-1};<br>H_2O_2: K_m = 0.1 mM, V_{max} = 0.14 × 10^{-8} M s^{-1}	3	Acid treatment of graphene oxide	24
Carboxyl-modified graphene oxide	TMB: K_m = 0.0237 mM, V_{max} = 3.45 × 10^{-8} M s^{-1};<br>H_2O_2: K_m = 3.99 mM, V_{max} = 3.85 × 10^{-8} M s^{-1}	4	$KMnO_4$ oxidation of graphene oxide	25
H@M	TMB: K_m = 10.9 mM, V_{max} = 8.98 × 10^{-8} M s^{-1};<br>H_2O_2: K_m = 0.068 mM, V_{max} = 6.07 × 10^{-8} M s^{-1}	5	Impregnation of hemin into MOF	26
Cubic Pt nanocrystal	N.A.	3.4	Solution reduction and heating	27
Rh NS	TMB: K_m = 0.264 mM, V_{max} = 12.56 × 10^{-8} M s^{-1}, k_{cat} = 8.2 × 10^4 s^{-1};<br>H_2O_2: K_m = 4.51 mM, V_{max} = 68.09 × 10^{-8} M s^{-1}, k_{cat} = 44.5 × 10^4 s^{-1};	4	Solution reduction and heating	28
MoS_2 NS	TMB: K_m = 0.525 mM, V_{max} = 5.16 × 10^{-8} M s^{-1};<br>H_2O_2: K_m = 0.0116 mM, V_{max} = 4.29 × 10^{-8} M s^{-1}	2-7.5	Solution-based exfoliation	29
$MoSe_2$ NS	TMB: K_m = 0.014 mM, V_{max} = 0.56 × 10^{-8} M s^{-1};<br>H_2O_2: K_m = 0.155 mM, V_{max} = 0.99 × 10^{-8} M s^{-1}	3.5	Liquid exfoliation method	30
WS_2 NS	TMB: K_m = 1.83 mM, V_{max} = 4.31 × 10^{-8} M s^{-1};<br>H_2O_2: K_m = 0.24 mM, V_{max} = 4.52 × 10^{-8} M s^{-1}	2-7	Commercially available	31
WSe_2 NS	TMB: K_m = 0.0433 mM, V_{max} = 1.43 × 10^{-8} M s^{-1};<br>H_2O_2: K_m = 19.53 mM, V_{max} = 2.22 × 10^{-8} M s^{-1}	3.5	Liquid exfoliation method	32

ZIF-67	TMB: K_m = 13.69 mM, V_{max} = 0.35 × 10^{-8} M s^{-1};<br>H_2O_2: K_m = 3.52 mM, V_{max} = 0.28 × 10^{-8} M s^{-1}	< 4	Heating and precipitation	33
Cu-MOF	TMB: K_m = 4.11 mM, V_{max} = 55.56 × 10^{-8} M s^{-1};<br>H_2O_2: K_m = 6.41 mM, V_{max} = 10.2 × 10^{-8} M s^{-1}	4	Solution reaction at room temperature	34
Fe_2O_3	NP: K_m = 36.82 mg/L, V_{max} = 1.36 mg/min, k_{cat} = 0.273 min^{-1}	8	Solution reaction at room temperature	35
2LFh	NP: K_m = 46.67 mg/L, V_{max} = 1.17 mg/min, k_{cat} = 0.234 min^{-1}	8	Solution reaction at room temperature	35
PdCu	TMB: K_m = 0.25 mM, V_{max} = 1.19 × 10^{-8} M s^{-1}, k_{cat} = 20.2 × 10^{-5} s^{-1};<br>H_2O_2: K_m = 3.05 mM, V_{max} = 6.25 × 10^{-6} M s^{-1}, k_{cat} = 10.61 × 10^{-2} s^{-1};	4	Microwave-assisted wet-chemical synthetic approach	36
Co_9S_8	TMB: K_m = 1.64 mM, V_{max} = 99 × 10^{-8} M s^{-1};<br>H_2O_2: K_m = 7.39 mM, V_{max} = 35 × 10^{-8} M s^{-1}	3	Heating and precipitation	37
$Cu(OH)_2$ SC	TMB: K_m = 2.448 mM, V_{max} = 44.83 × 10^{-8} M s^{-1};<br>H_2O_2: K_m = 0.199 mM, V_{max} = 42.51 × 10^{-8} M s^{-1}	4.5	Solution reaction at room temperature	38
Ru frame	TMB: K_m = 0.0603 mM, V_{max} = 13.4 × 10^{-8} M s^{-1};<br>H_2O_2: K_m = 318 mM, V_{max} = 7.41 × 10^{-8} M s^{-1}	0-12	Heating and precipitation	39
FePt-Au HNP	TMB: K_m = 0.445 mM, V_{max} = 24.67 × 10^{-8} M s^{-1};<br>H_2O_2: K_m = 0.0185 mM, V_{max} = 0.6894 × 10^{-8} M s^{-1}	4	Hydrothermal method	40
PtAg-MoS_2	TMB: K_m = 25.71 mM, V_{max} = 7.29 × 10^{-8} M s^{-1};<br>H_2O_2: K_m = 0.386 mM, V_{max} = 3.22 × 10^{-8} M s^{-1}	4	Heating and precipitation	41
PtPd NP	TMB: K_m = 1.78 mM, V_{max} = 36.4 × 10^{-8} M s^{-1};<br>H_2O_2: K_m = 0.053 mM, V_{max} = 9.26 × 10^{-8} M s^{-1}	9.5	Reduction and mild heating	42
Cu-hemin	TMB: K_m = 1.42 mM, V_{max} = 26.22 × 10^{-8} M s^{-1};<br>H_2O_2: K_m = 2.18 mM, V_{max} = 116 × 10^{-8} M s^{-1}	6	Reduction and mild heating	43
PS@Au@PB	TMB: K_m = 1.22 mM, V_{max} = 59 × 10^{-8} M s^{-1};<br>H_2O_2: K_m = 0.17 mM, V_{max} = 38.9 × 10^{-8} M s^{-1}	5.2	Solution reaction at room temperature	44
Cu NC	TMB: K_m = 0.648 mM, V_{max} = 5.96 × 10^{-8} M s^{-1};<br>H_2O_2: K_m = 29.16 mM, V_{max} = 4.22 × 10^{-8} M s^{-1}	6	Solution reaction and mild heating	45

Table S2. BET (Brunauer-Emmett-Teller) characterizations of γ-Al_2O_3 support and synthesized $FeOCl/Al_2O_3$.

		γ-Al_2O_3	$FeOCl/Al_2O_3$
BET Surface Area		883.9819 m^2/g	212.7531 m^2/g
Pore Volume	BJH Adsorption cumulative volume of pores between 1.7 nm and 300 nm width	1.396243 cm^3/g	0.577339 cm^3/g
	BJH Desorption cumulative volume of pores between 1.7 nm and 300 nm width	1.401154 cm^3/g	0.556347 cm^3/g
Pore Size	Adsorption average pore diameter (4V/A by BET)	5.92993 nm	10.94665 nm
	BJH Adsorption average pore width (4V/A)	7.8207 nm	8.7791 nm
	BJH Desorption average pore width (4V/A)	7.0510 nm	9.1477 nm

Table S3. Quantification methods of selected organic compounds.

Substrate	Detection method	Mobile phase	Ratio	Flow rate (mL/min)	Detection wavelength
Ibuprofen	HPLC	methanol/water	68/32	0.5	228 nm
Atrazine	HPLC	methanol/water	60/40	0.5	228 nm
Carbamazepine	HPLC	methanol/water	60/40	0.5	285 nm
4-Chlorophenol	HPLC	acetonitrile/water	30/70	0.5	225 nm
4-Nitrophenol	HPLC	acetonitrile/water	60/40	0.5	280 nm
Rhodamine B	UV-Vis spectrometer	—	—	—	554 nm
Reactive blue 19	UV-Vis spectrometer	—	—	—	592 nm
Orange II	UV-Vis spectrometer	—	—	—	485 nm

Table S4. Characterizations of field water samples.

Water sample	Water type	Total organic carbon (mg/L)	Dissolved oxygen (mg/L)	pH
Sample 1	Lake water	21.4	8.38	8.51
Sample 2	Lake water	115.5	7.49	8.29
Sample 3	Underground water	87.2	7.92	7.31
Sample 4	Underground water	158.7	7.21	7.78

Supplemental References:

1. De Smedt, F., and Wierenga, P.J. (1984). Solute transfer through columns of glass beads. Water Resources Res. *20*, 225-232.

2. Acar, Y.B., and Alshawabkeh, A.N. (1993). Principles of electrokinetic remediation. Environ. Sci. Technol. *27*, 2638–2647.

3. Davis, E.M., and Davis, R.J. (2003). Fundamentals of chemical reaction engineering. (McGraw-Hill Higher Education).

4. Hojabri, S., Rajic, L., and Alshawabkeh, A.N. (2018). Transient reactive transport model for physico-chemical transformation by electrochemical reactive barriers. J. Hazard. Mater. *358*, 171-177.

5. Paz-García, J.M., Villén-Guzmán, M., García-Rubio, A., Hall, S., Ristinmaa, M., and César, G.L. (2016). A coupled reactive-transport model for electrokinetic remediation. In Electrokinetics Across Disciplines and Continents, Ribeiro, A.B., Mateus, E.P., and Couto, N. (Springer International Publishing), pp. 251–278.

6. Steefel, C.I., and Lasaga, A.C. (1994). A coupled model for transport of multiple chemical species and kinetic precipitation/dissolution reactions with application to reactive flow in single phase hydrothermal systems. Am. J. Sci. *294*, 529-592.

7. Parkhurst, D.L., and Appelo, C.A.J. (2013). PHREEQC (version 3)-a computer program for speciation, batch-reaction, one-dimensional transport, and inverse geochemical calculations. In Modeling Techniques, p. 497.

8. Nardi, A., Idiart, A., Trinchero, P., De Vries, L. M., and Molinero, J. (2014). Interface COMSOL-PHREEQC (iCP), an efficient numerical framework for the solution of coupled multiphysics and geochemistry. Comput. Geosci. *69*, 10–21.

9. Versteeg, H.K., and Malalasekera, W. (2007). An introduction to computational fluid dynamics: the finite volume method. (Pearson Education).

10. Gao, L., Zhuang, J., Nie, L., Zhang, J., Zhang, Y., Gu, N., Wang, T., Feng, J., Yang, D., Perrett, S., and Yan, X. (2007). Intrinsic peroxidase-like activity of ferromagnetic nanoparticles. Nat. Nanotechnol. *2*, 577-583.

11. Deng, H., Li, X., Peng, Q., Wang, X., Chen, J., and Li. Y. (2005). Monodisperse magnetic single-crystal ferrite microspheres. Angew. Chem. Int. Ed. *44*, 2782–2785.

12. Ma, M., Wu, Y., Zhou, J., Sun, Y., Zhang, Y., and Gu, N. (2004). Size dependence of specific power absorption of Fe_3O_4 particles in AC magnetic field. J. Magn. Magn. Mater. *268*, 33–39.

13. Zeb, A., Sahar, S., Qazi, U.Y., Odda, A.H., Ullah, N., Liu, Y., Qazi, I.A., and Xu, A.W. (2018). Intrinsic peroxidase-like activity and enhanced photo-Fenton reactivity of iron-substituted polyoxometallate nanostructures. Dalton Trans. *47*, 7344-7352.

14. Wang, J., Han, D., Wang, X., Qi, B., and Zhao, M. (2012). Polyoxometalates as peroxidase mimetics and their applications in H_2O_2 and glucose detection. Biosens. Bioelectron. *36*, 18-21.

15. Sun, C., Chen, X., Xu, J., Wei, M., Wang, J., Mi, X., Wang, X., Wu, Y., and Liu, Y. (2013). Fabrication of an inorganic–organic hybrid based on an iron-substituted polyoxotungstate as a peroxidase for colorimetric immunoassays of H_2O_2 and cancer cells. J. Mater. Chem. A *1*, 4699-4705.

16. Qu, K., Shi, P., Ren, J., and Qu, X. (2014). Nanocomposite incorporating V_2O_5 nanowires and gold nanoparticles for mimicking an enzyme cascade reaction and its application in the detection of biomolecules. Chem.: Eur. J. *20*, 7501-7506.

17. Asati, A., Santra, S., Kaittanis, C., Nath, S., and Perez, J.M. (2009). Oxidase-like activity of polymer-coated cerium oxide nanoparticles. Angew. Chem. Int. Ed. *121*, 2344-2348.

18. Mu, J., Wang, Y., Zhao, M., and Zhang, L. (2012). Intrinsic peroxidase-like activity and catalase-like activity of Co_3O_4 nanoparticles. Chem. Comm. *48*, 2540-2542.

19. Peng, Y., Wang, Z., Liu, W., Zhang, H., Zuo, W., Tang, H., Chen, F., and Wang, B. (2015). Size-and shape-dependent peroxidase-like catalytic activity of $MnFe_2O_4$ nanoparticles and their applications in highly efficient colorimetric detection of target cancer cells. Dalton Trans. *44*, 12871-12877.

20. Wang, Q., Ma, K., Yu, Z., Ding, J., Hu, Q., Liu, Q., Sun, H., Wen, D., Liu Q., and Kong, J. (2018). The peroxidase-like catalytic activity of ferrocene and its application in the biomimetic synthesis of microsphere polyaniline. New J. Chem. *42*, 13536-13540.

21. Liu, Y., Zhu, G., Yang, J., Yuan, A., and Shen, X. (2014). Peroxidase-like catalytic activity of Ag_3PO_4 nanocrystals prepared by a colloidal route. PloS one *9*, e109158.

22. Kim, M. C., Lee, D., Jeong, S.H., Lee, S.Y., and Kang, E. (2016). Nanodiamond–gold nanocomposites with the peroxidase-like oxidative catalytic activity. ACS Appl. Mater. Interfaces *8*, 34317-34326.

23. Zheng, H., Liu, C., Zeng, X., Chen, J., Lü, J., Lin, R., Cao, R., Lin, Z., and Su, J. (2018). MOF-808: A metal–organic framework with intrinsic peroxidase-like catalytic activity at neutral pH for colorimetric biosensing. Inorg. Chem. *57*, 9096-9104.

24. Lin, L., Song, X., Chen, Y., Rong, M., Zhao, T., Wang, Y., Jiang, Y., and Chen, X. (2015). Intrinsic peroxidase-like catalytic activity of nitrogen-doped graphene quantum dots and their application in the colorimetric detection of H_2O_2 and glucose. Anal. Chim. Acta *869*, 89-95.

25. Song, Y., Qu, K., Zhao, C., Ren, J., and Qu, X. (2010). Graphene oxide: intrinsic peroxidase catalytic activity and its application to glucose detection. Adv. Mater. *22*, 2206-2210.

26. Qin, F., Jia, S., Wang, F., Wu, S., Song, J., and Yong Liu. (2013). Hemin@metal–organic framework with peroxidase-like activity and its application to glucose detection. Catal. Sci. Technol. *3*, 2761-2768.

27. Ma, M., Zhang, Y., and Gu, N. (2011). Peroxidase-like catalytic activity of cubic Pt nanocrystals. Colloids Surf. A *373*, 6-10.

28. Cai, S., Xiao, W., Duan, H., Liang, X., Wang, C., Yang, R., and Li, Y. (2018). Single-layer Rh nanosheets with ultrahigh peroxidase-like activity for colorimetric biosensing. Nano Res. *11*, 6304-6315.

29. Lin, T., Zhong, L., Guo, L., Fu, F., and Chen, G. (2014). Seeing diabetes: visual detection of glucose based on the intrinsic peroxidase-like activity of MoS_2 nanosheets. Nanoscale *6*, 11856-11862.

30. Wu, X., Chen, T., Wang, J., and Yang, G. (2018). Few-layered $MoSe_2$ nanosheets as an efficient peroxidase nanozyme for highly sensitive colorimetric detection of H_2O_2 and xanthine. J. Mater. Chem. B *6*, 105-111.

31. Lin, T., Zhong, L., Song, Z., Guo, L., Wu, H., Guo, Q., Chen, Y., Fu, F., and Chen, G. (2014). Visual detection of blood glucose based on peroxidase-like activity of WS_2 nanosheets. Biosens. Bioelectron. *62*, 302-307.

32. Chen, T.M., Wu, X.J., Wang, J.X., and Yang, G.W. (2017). WSe_2 few layers with enzyme mimic activity for high-sensitive and high-selective visual detection of glucose. Nanoscale *9*, 11806-11813.

33. Wang, S., Xu, D., Ma, L., Qiu, J., Wang, X., Dong, Q., Zhang, Q., Pan, J., and Liu, Q. (2018). Ultrathin ZIF-67 nanosheets as a colorimetric biosensing platform for peroxidase-like catalysis. Anal. Bioanal. Chem. *410*, 7145-7152.

34. Wang, C., Gao, J., and Tan, H. (2018). Integrated antibody with catalytic metal–organic framework for colorimetric immunoassay. ACS Appl. Mater. Interfaces *10*, 25113-25120.

35. Pariona, N., Herrera-Trejo, M., Oliva, J., and Martinez, A.I. (2016). Peroxidase-like activity of ferrihydrite and hematite nanoparticles for the degradation of methylene blue. J. Nanomater. *2016*, 3427809.

36. He, Y., Niu, X., Li, L., Li, X., Zhang, W., Zhao, H., Lan, M., Pan, J., and Zhang. X. (2018). Microwave-assisted fabrication of bimetallic PdCu nanocorals with enhanced peroxidase-like activity and efficiency for thiocyanate sensing. ACS Appl. Nano Mater. *1*, 2397-2405.

37. Mu, J., Li, J., Zhao, X., Yang, E.C., and Zhao, X.J. (2018). Novel urchin-like Co_9S_8 nanomaterials with efficient intrinsic peroxidase-like activity for colorimetric sensing of copper(II) ion. Sensor. Actuat. B: Chem. *258*, 32-41.

38. Cai, R., Yang, D., Peng, S., Chen, X., Huang, Y., Liu, Y., Hou, W., Yang, S., Liu, Z., and Tan, W. (2015). Single nanoparticle to 3D supercage: framing for an artificial enzyme system. J. Am. Chem. Soc. *137*, 13957-13963.

39. Ye, H., Mohar, J., Wang, Q., Catalano, M., Kim, M.J., and Xia, X. (2016). Peroxidase-like properties of ruthenium nanoframes. Sci. Bull. *61*, 1739-1745.

40. Ding, Y., Yang, B., Liu, H. Liu, Z., Zhang, X., Zheng, X., and Liu. Q. (2018). FePt-Au ternary metallic nanoparticles with the enhanced peroxidase-like activity for ultrafast colorimetric detection of H_2O_2. Sensor. Actuat. B: Chem. *259*, 775-783.

41. Cai, S., Han, Q., Qi, C., Lian, Z., Jia, X., Yang, R., and Wang, C. (2016). $Pt_{74}Ag_{26}$ nanoparticle-decorated ultrathin MoS_2 nanosheets as novel peroxidase mimics for highly selective colorimetric detection of H_2O_2 and glucose. Nanoscale *8*, 3685-3693.

42. Jiang, T., Song, Y., Du, D., Liu, X., and Lin, Y. (2016). Detection of p53 protein based on mesoporous Pt–Pd nanoparticles with enhanced peroxidase-like catalysis. ACS Sensors *1*, 717-724.

43. Liu, F., He, J., Zeng, M., Hao, J., Guo, Q., Song, Y. and Wang, L. (2016). Cu–hemin metal-organic frameworks with peroxidase-like activity as peroxidase mimics for colorimetric sensing of glucose. J. Nanopart. Res. *18*, 106.

44. Zhang, X.Z., Zhou, Y., Zhang, W., Zhang, Y., and Gu, N. (2016). Polystyrene@Au@prussian blue nanocomposites with enzyme-like activity and their application in glucose detection. Colloids Surf. A *490*, 291-299.

45. Hu, L., Yuan, Y., Zhang, L., Zhao, J., Majeed, S., Xu, G. (2013). Copper nanoclusters as peroxidase mimetics and their applications to H_2O_2 and glucose detection. Anal. Chim. Acta *762*, 83-86.
